# The Prognostic and Therapeutic Potential of DNA Damage Repair Pathway Alterations and Homologous Recombination Deficiency in Lung Cancer

**DOI:** 10.3390/cancers14215305

**Published:** 2022-10-28

**Authors:** Karam Khaddour, Manuel Felipe Fernandez, Marsel Khabibov, Airat Garifullin, Danielle Dressler, Iuliia Topchu, Jyoti D. Patel, Frank Weinberg, Yanis Boumber

**Affiliations:** 1 Division of Hematology and Oncology, Department of Medicine, University of Illinois Chicago, Chicago, IL 60612, USA; 2 Robert H. Lurie Comprehensive Cancer Center, Division of Hematology/Oncology, Feinberg School of Medicine, Northwestern University, Chicago, IL 60611, USA; 3 I. M. Sechenov First Moscow State Medical University, 119992 Moscow, Russia; 4 P. Hertsen Moscow Oncology Research Institute, National Medical Research Radiological Centre of the Ministry of Health of the Russian Federation, 125284 Moscow, Russia; 5 Institute of Fundamental Medicine and Biology, Kazan (Volga Region) Federal University, 420012 Kazan, Russia

**Keywords:** non-small cell lung cancer, small cell lung cancer, DNA-damage repair, homologous recombination deficiency, PARP

## Abstract

**Simple Summary:**

DNA-damage repair (DDR) gene alterations are a hallmark for cancer. The exploitation of DDR alterations has improved outcomes in breast, ovarian, pancreatic, and prostate cancers. However, little is known about the role of DDR alterations, leading to a homologous recombination deficiency (HRD) state in lung cancer. In this review, we discuss the existing literature examining the role of DDR alterations as both predictive biomarkers of response to therapy and as therapeutic targets in lung cancer.

**Abstract:**

Lung cancer remains the second most commonly diagnosed cancer worldwide and the leading cause of cancer-related mortality. The mapping of genomic alterations and their role in lung-cancer progression has been followed by the development of new therapeutic options. Several novel drugs, such as targeted therapy and immunotherapy, have significantly improved outcomes. However, many patients with lung cancer do not benefit from existing therapies or develop progressive disease, leading to increased morbidity and mortality despite initial responses to treatment. Alterations in DNA-damage repair (DDR) genes represent a cancer hallmark that impairs a cell’s ability to prevent deleterious mutation accumulation and repair. These alterations have recently emerged as a therapeutic target in breast, ovarian, prostate, and pancreatic cancers. The role of DDR alterations remains largely unknown in lung cancer. Nevertheless, recent research efforts have highlighted a potential role of some DDR alterations as predictive biomarkers of response to treatment. Despite the failure of PARP inhibitors (main class of DDR targeting agents) to improve outcomes in lung cancer patients, there is some evidence suggesting a role of PARP inhibitors and other DDR targeting agents in benefiting a distinct subset of lung cancer patients. In this review, we will discuss the existing literature on DDR alterations and homologous recombination deficiency (HRD) state as predictive biomarkers and therapeutic targets in both non-small cell lung and small cell lung cancer.

## 1. Introduction

Lung cancer represents a major healthcare burden despite the introduction of several novel effective medications that improves outcomes [1]. Globally, lung cancer is the leading cause of death for men (1.2 million deaths per year), and the second leading cause of death for women (576,100) among all cancers [2]. Lung cancer is divided histologically and clinically into non-small cell lung cancer (NSCLC) (approximately 85% of newly diagnosed lung cancer) and small cell lung cancer (SCLC) (approximately 15%). NSCLC is further classified into subcategories including adenocarcinoma (LUAD) and squamous cell lung carcinoma (LUSC), which have distinct molecular features, treatment approaches, and different prognoses. The treatment of NSCLC has been revolutionized with the introduction of several novel therapeutics such as targeted therapy and immunotherapy. Targeted therapy is indicated in patients with metastatic lung cancer whose tumors harbor an oncogenic driver mutation. However, approximately a third of patients with lung adenocarcinoma (LUAD) and the majority of LUSC and SCLC patients do not have targetable oncogenic driver mutations [3,4,5]. Moreover, several oncogenic driver mutations remain undruggable or only have medications approved in the second-line setting such as with *KRAS^G12C^* inhibitors [6]. Patients with NSCLC without identifiable oncogenic driver alterations can be treated with either chemotherapy or chemoimmunotherapy, depending on the cancer subtype and programmed death-ligand 1 (PD-L1) expression. Despite the improvement in overall survival (OS) with the integration of immune checkpoint inhibitors (ICI) in early-stage and metastatic lung cancer, the benefit in OS appears to be driven by a subset of patients whose tumors have a high PD-L1 expression ≥ 50% [7,8]. Patients with PD-L1 expression levels 0% and 1–49% appear to have variable outcomes with modest benefit with immunotherapy alone, which is moderately improved with the use of chemoimmunotherapy [9]. As such, there is an unmet need to explore potential new effective therapeutic approaches to improve outcomes in NSCLC. Moreover, SCLC, which is considered a recalcitrant tumor, represents a challenge for patients and physicians given the lack of effective therapeutics and poor outcomes relative to other cancers. Recent advances in the ability to map genomic alterations in lung cancer have led to the identification of relevant targetable pathways and provided the means to study several biomarkers, which could help to select patients for specific treatments.

**Figure 1:**
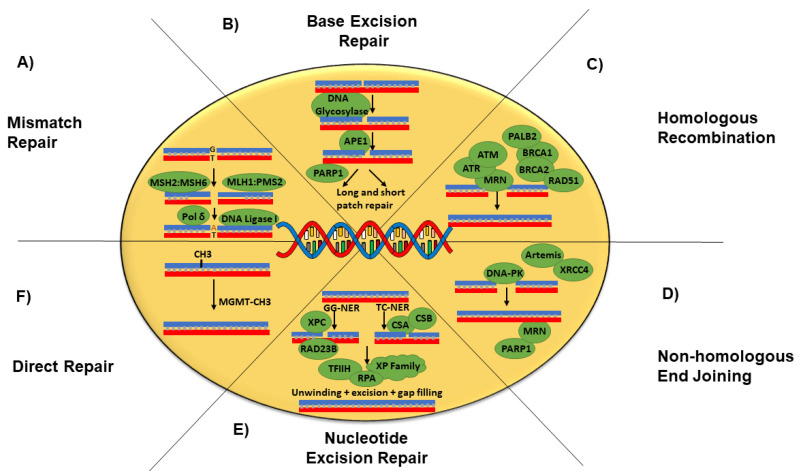
Summary of the six main mechanisms responsible for the DDR. (**A**) Mismatch repair: At the beginning of the process of the mismatch repair, mispaired bases are recognized by the sliding clamp MutSα homolog (MSH2:MSH6). Subsequently, the MLH1:PMS2complex is recruited for the incision, followed by the excision of the damage by exonuclease-1, resulting in a gap, which is then filled by Polδ and DNA ligase I. (**B**) BER: This pathway is responsible for the removal of small covalent modifications. These lesions are recognized by one of the specific DNA glycosylases and then hydrolyzed by AP-endonuclease (APE). PARP1 is recruited by nicked DNA due to APE1 and aid in DNA repair. If a single nucleotide is damaged, then the shortpatch BER pathway repairs it. The long-patch BER pathway is responsible for the repair of a lesion of at least two nucleotides. (**C**) Homologous recombination: This pathway is involved in the repair of the double-strand breaks and is carried out in late S and G2 phases when the homologous sister chromatid can be used as a template. In this process, ATM facilitates the activity of MRN complex at the site of DNA damage. The MRN complex then resects DNA from the dsDNA break site and the DNA strand is digested. As a result, single-stranded DNA is produced and RPA binds to it. BRCA1 and BRCA2 proteins (linked by PALB2 protein) control Rad51 that facilitates the search for a homologous DNA sequence and the formation of a D-loop. DNA polymerase eta finishes this process. (**D**) NHEJ: This pathway is involved in DSB repair but does not require a homologous template. Two free DNA ends are bound by Ku70/Ku80 heterodimer, DNA-PK is then utilized, and an active catalytic complex is formed. Subsequently, artemis endonuclease is also recruited for the endonucleolytic cleavage, then the Ligase IV/XRCC4 complex ligates DNA ends. A sub-pathway of NHEJ, which is an alternative end-joining, utilizes the Pol θ, PARP1 and MRN complex for DNA repair. (**E**) NER: This pathway is divided into two sub-pathways based on the DNA damage recognition step. Global genome nucleotide excision repair (GG-NER) detects and eliminates bulky DNA excisions throughout the genome, including non-transcribed regions and silent chromatin. In this pathway, XPC-RAD23B complex recognizes DNA lesions and recruits downstream factors. Transcription-coupled NER (TC-NER) operates when a transcribed DNA strand is damaged, limiting transcription activity. TC-NER is triggered by RNAPIIo-bound Cockayne Syndrome Group B (CSB) protein, which recruits CSA via the CSA-interaction motif (CIM). Once recruited, CSA facilitates UVSSA’s association with stalled RNA Pol II (RNA P IIo). UVSSA is the key factor that recruits the TFIIH complex in response to CSB and CSA. TC-NER and GG-NER use the same mechanism after detecting damage. The TFIIH complex unwinds the DNA helix to obtain access to the pre-incision complex (XP family), allowing for RPA to be recruited. The damaged DNA is then excised, aided by XPG and the endonucleases excision repair cross-complementing enzyme group 1 (CPF-ERCC1). Then, the DNA gap is refilled by PCNA and RPC, followed by backbone repair via DNA ligase I. (**F**) Direct repair: The presence of the alkyl group at the O6 position of guanine causes G:C to A:T transitions. The direct repair functions through the removal of the alkyl group at the site of lesion by the enzyme O6-methylguanine DNA methyltransferase (MGMT), thus restoring the proper nucleotide.

DNA-damage repair (DDR) pathways are essential for cellular integrity and high-fidelity genome repair that results from exogenous and endogenous DNA insults. They consist of multiple genes that are responsible for maintaining genomic stability by removing damaged DNA in a substrate-specific manner. Alterations in DDR pathways are an important hallmark for tumorigenesis, cancer progression and prognosis [10]. The analysis of The Cancer Genome Atlas (TCGA) in 33 cancer types demonstrated that mutations with loss of heterozygosity in various tumor types were observed in one third of DDR genes [10]. DDR pathways can be functionally classified into nucleotide excision repair (NER), base excision repair (BER), direct repair (DR), DNA double-stranded break repair including homologous recombination (HR) and non-homologous end joining (NHEJ), and mismatch repair (MMR) [11] (Figure 1). These pathways work in a highly orchestrated manner to maintain genomic stability and prevent the accumulation of mutations leading to senescence, apoptosis or clonal cancerous evolution. NER is an important DNA repair pathway that consists of two sub-pathways (global genomic NER (GG-NER) and transcription-coupled NER (TC-NER)) [12]. DNA alterations that are primarily targeted by NER include bulky DNA adducts caused by UV light and chemotherapeutic agents such as platinum agents inducing interstrand crosslinks [12]. MMR is responsible for the repair of base substitution mismatches, insertion–deletion mismatches, and DNA crosslinks. BER is responsible for the removal of small covalent modifications of DNA bases and requires five enzymatic activities: glycosylase, endonuclease, lyase, polymerase, and ligase. It includes two sub-pathways: short-patch (i.e., single-nucleotide) and long-patch BER (>1 nucleotide) [13]. The endonuclease activity also leads to the single-strand break repair pathway, which stimulates the poly(ADP-ribose) activity of PARP1, which will be discussed later [11]. DNA double-strand breaks (DSBs) are induced by ionizing radiation, chemotherapeutic agents, faulty DNA replication, and oxidative stress. Two main pathways involved in the repair of DSBs include HR and NHEJ. Both BRCA1 and BRCA2 proteins interact with a variety of proteins involved in the HR pathway and appear to be essential to the process, acting at different stages of DSB repair [14].

Defects in the function of cell regulatory checkpoints can promote cancer growth through the accumulation of deleterious mutations [15]. The impact of DDR gene alterations on cancer formation is important in patients with hereditary cancer syndromes such as germline *BRCA1/2* pathologic mutation carriers who have a higher relative risk incidence of breast, ovarian, uterine, and pancreatic cancers. However, DDR-deficient tumors still require intact mechanisms to repair the DNA damage necessary for survival and shift dependence to other intact DNA repair pathways. As such, targeting these dependent pathways in DDR-deficient cancer cells leads to a synthetically lethal effect that can halt the growth of cancer cells [16]. This has been studied extensively in the preclinical and clinical setting in cancers harboring *BRCA1/2* mutations, which induce homologous recombination deficiency (HRD), leading to a reliance on other compensatory DDR pathways such as BER and NHEJ [17]. Alterations in homologous recombination pathway genes have been extensively studied in cancer, due to their high suitability for cancer hereditary syndromes in pathologic *BRCA1/2* germline mutation carriers. Most of the novel cancer therapies that are being developed to target DDR pathway involve inhibitors of homologous recombination genes. Therefore, HRD-scoring algorithms have been developed using different assays to allow for the quantification of the degree of genomic instability as multiple genes, in addition to *BRCA1/*2, play important roles in DNA repair. Several HRD scores were developed, which rely on algorithms pertaining to loss of heterozygosity (LOH), number of telomeric allelic imbalances (NtAI), and large-scale transitions (LST) [18,19,20]. In clinical trials, HRD-deficient ovarian and breast cancers due to *BRCA1/2* germline mutations rely on poly(ADP-ribose) polymerase (PARP) to repair single and double-stranded breaks. Therefore, targeting PARP enzymes with specific inhibitors leads to synthetic lethality and cancer cell death. The FDA and EMA have approved several PARP inhibitors (PARPi) that demonstrated clinical efficacy and acceptable safety in *BRCA1/2* mutated breast and ovarian cancers [21,22]. The clinical efficacy was evident in other cancer types due to *BRCA1/2* or other deleterious mutations in the HR pathway (such as *ATM*), specifically pancreatic and prostate cancers [23]. The activity of PARPi in certain cancers with deficient homologous recombination repair ability (mainly due to *BRCA1/2* mutations) was the first clinical demonstration of the role of exploiting HR alterations to improve outcomes and prolong survival in cancer patients. The benefit of targeting homologous recombination pathway alterations has been limited to a few cancers with specific molecular signatures due to dysfunction of the HR pathway (*BRCA1/2*, *ATM* and HRD state). The impact of possible DDR alterations (which entails more than 350 candidate genes) remains insufficiently explored in oncology. Recently, the term BRCAness was developed to refer to cancers that have the phenotype of *BRCA1/2* mutations due to non-BRCA DDR alterations, epigenetic modulation and post translational modification [16].

Research is ongoing regarding the impact of DDR alterations on cancer formation, progression and therapeutic potential in other cancer types, including lung cancer. The fact that alterations in DDR genes are detected in a third of cancers raises several questions regarding the role of such deficiencies in lung cancer. This is of importance as several drugs that target DDR alterations are currently being developed and studied. These include PARP inhibitors, inhibitors of ATR and ATM, inhibitors of CHK1/2, inhibitors of WEE1 and their combinations, including with one another, chemotherapy, targeted therapy, or immunotherapy [16]. Smoking, which is a major risk factor in lung cancer, induces DNA damage, promoting the activation of several repair mechanisms that could provide a rationale for targeting DDR defects in selected lung cancer patients [24,25,26]. However, despite the evidence that smoking-related lung cancer is associated with a higher somatic tumor mutation burden, it remains unclear if specific DDR gene alterations are more common in lung cancer that arises in smokers.

In this review, we discuss the current and previous literature regarding the role of DDR alterations (mainly HR) and HRD status in NSCLC and SCLC. We will discuss the frequency and pathological impact of DDR alterations in both types of lung cancer. Next, we will discuss the predictive and prognostic potential of DDR alterations as biomarkers for NSCLC and SCLC. Additionally, we will review the literature on the clinical relevance of targeting DDR alterations (mainly HR pathway) and the HRD phenotype of lung cancers.

## 2. Frequency of DDR Alterations in Lung Cancer

Heeke et al. studied the prevalence of HR-DDR deficiency across 21 tumor types including the following genes (*ARID1A*, *ATM*, *ATRX*, *BAP1*, *BARD1*, *BLM*, *BRCA1/2*, *BRIP1*, *CHEK1/2*, *FANCA/C/D2/E/F/G/L*, *MRE11A*, *NBN*, *PALB2*, *RAD50*, *RAD51*, *RAD51B*, and *WRN*). Frequent HR-DDR mutations were observed in 17.4% of tumors. The overall frequency of HR-DDR mutations was 14.2% (95%CI: 13.0 to 15.5) in NSCLC (*n* = 8119), and 10.0% (95%CI: 7.4 to 13.2) in neuroendocrine/SCLC (*n* = 1498). The most mutated genes in NSCLC included *ARID1A* (4.61%), *ATM* (3.48%), *BRCA2* (0.83%), *CHEK2* (1.23%), *BRCA1* (0.83%). In SCLC, common DDR mutations were observed in *ARID1A* (4.04%), *ATRX* (2.02%), *ATM* (1.34%), *CHEK2* (1.11%) [27].

Knijnenburg et al. analyzed DDR deficiency in 33 cancer types across 9125 cancer samples from TCGA database. They performed an analysis of 276 genes, which encompassed all major DNA repair pathways: 208 genes were annotated to one or more specific DDR pathway, with an additional 68 genes being annotated to key DDR-related pathways. In LUSC genes involved in HR were altered in 16%, DR—in 16%, DS—in 15%, MMR—12%. In LUAD, genes were altered in HR—18%, DS—15%, DR—15% [10].

**Figure 2:**
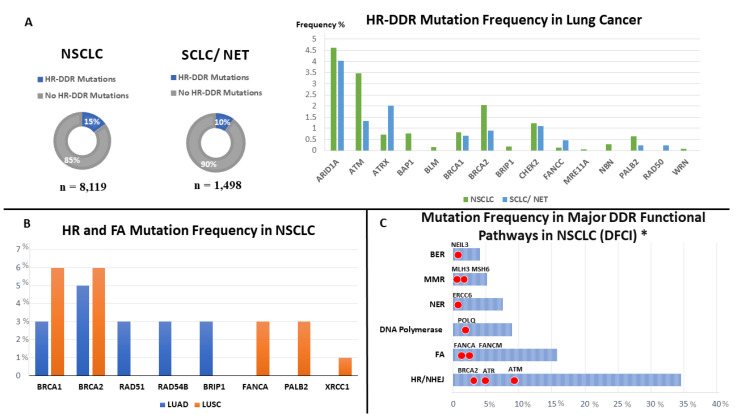
Reported frequencies of DDR alterations in NSCLC and SCLC. (**A**) Frequency of HR-DDR mutations from Caris cohort in NSCLC and SCLC represented in pie charts and, to the right of the panel, the reported frequencies of most common mutated HR-DDR genes in NSCLC (green) and SCLC (blue). (**B**) Selected reported frequencies in lung adenocarcinoma (LUAD) (*n* = 230) and lung squamous cell carcinoma (LUSC) (*n* = 178) from the TCGA database in NSCLC. (**C**) Mutation frequency in major DDR functional pathways in NSCLC from the Dana-Farber Cancer Institute cohort (*n* = 266), with blue bar charts representing frequency of the major DDR pathway mutations, and red dots representing specific highly mutated genes within the major DDR pathway. *: This cohort include metastatic non-small cell lung cancer patients treated with PD-(L)1 inhibitors.

An additional study identified somatic mutations in 188 human lung adenocarcinomas with an overall mutation frequency of 14.5% for *ATM,* which is a cell-cycle checkpoint kinase [28]. Parry et al. systematically tested whether germline mutations in eight candidate genes could be risk factors for lung adenocarcinoma. They found 14 pathogenic mutations in five genes in 555 lung adenocarcinoma cases, with a frequency of 2.5%. Among DDR genes, *ATM* had the highest level of mutations (50%), followed by *BRCA2* (7%). This suggests that, like other solid tumors, lung adenocarcinoma has a small subset of patients with inherited susceptibility due to DDR deficiencies [29]. Alterations in MMR genes were identified in a subset of mostly untreated NSCLC patients (both adenocarcinoma and LUSC) that displayed low expression of the *MLH1* gene [30]. However, the presence of microsatellite instability is rare in both NSCLC (0.31%) and SCLC (0.61%) [31]. In another study, Waqar et al. identified somatic mutations in HR and Fanconi Anemia (FA) pathways in NSCLC using the cBioportal from TCGA (230 LUAD and 178 LUSC). The investigators found homozygous deletion mutation in *RAD51* (3%) in LAUD and *XRCC1* (1%) in LUSC. Other mutations that were identified included *BRCA1/2*, *PALB2*, *BRIP1*, *RAD51D*, *RAD54L* and *FANCA* [32]. Similarly, a patient cohort at the Dana Farber Cancer Institute, including 266 patients with advanced NSCLC who were treated with PD-(L)1 inhibitors, demonstrated the presence of pathologic DDR alterations in 49.6% of patients, with the most common mutations involving *ATM*, *ATR*, *BRCA2*, *POLQ,* and *RAD50* [33] (Figure 2).

In SCLC, key driver mutations are loss of *TP53* and *RB1* tumor-suppressor genes, which are present in approximately 80–90% of patients [34,35]. In addition, as outlined in the study by Heek et al., 10% of neuroendocrine/SCLC tumor samples harbor HR-DDR alterations [27]. A study of 166 patients with SCLC found that 47.8% had genomic alterations involving either double- or single-stranded repair genes [36]. Of interest, the proteomic profiling of 34 SCLC cell lines demonstrated the overexpression of DDR proteins including PARP, ATR, CHK1/2 and ATM, which was shown to provide a survival advantage for cancer cells [37].

Therefore, the presence of mutations and perhaps changes in expression in key DDR genes in lung cancer provides a rationale for further research on the biological implications of these alterations on cancer cell survival. The defective DDR pathways that lead cancer cells to rely on other compensatory DDR mechanisms could be further explored as a therapeutic target. However, more research is needed given the limited understanding of the biological consequences of DDR alterations in lung cancer.

## 3. Prognostic and Predictive Impact of Alterations in DNA Damage Repair Genes in NSCLC

Platinum therapy has long been the backbone of chemotherapy for NSCLC, although it has a historically modest survival benefit in early-stage and metastatic disease [38,39]. A 2006 study by Olaussen et al. sought to describe a validated predictor of survival benefit with an adjuvant cisplatin regimen [40]. As nucleoside excision repair has been understood to confer resistance to platinum-based chemotherapy, the investigators compared clinical outcomes in patients whose resected tumors had a detectable expression of ERCC1 (a critical mediator of nucleotide excision repair) to patients who did not have detected ERCC1 [41,42]. The authors found that patients with ERCC1+ tumors did not derive any significant survival benefit from adjuvant cisplatin compared to the control arm, which suggested a predictive role of ERCC1 status to cisplatin sensitivity [40]. While these results were encouraging, subsequent analyses raised the concern that the available antibodies could not always reliably select for the functional ERCC1 isoform, thus limiting their utility as a predictive tool in the clinical setting [43,44]. This study has provided a proof-of-concept on the possible role of DDR pathway alterations as a possible predictor of response to lung cancer treatment.

The role of other DDR pathway alterations in predicting NSCLC response to platinum therapy has also been assessed. Despite the association between *BRCA* mutations with platinum sensitivity in other tumor types, alterations in *BRCA* remain controversial regarding their predictive ability in NSCLC treated with platinum-based therapy [45,46,47]. For example, a retrospective study of tumor specimens from 100 NSCLC patients treated with platinum-based therapy assessed the effect of low BRCA1 expression on response and survival. The authors found that the low expression of *BRCA1* detected by RT-qPCR was associated with higher response rates and longer progression-free survival (PFS) and OS [48]. However, this study did not investigate *BRCA1* alterations on the genomic or epigenomic levels. In contrast, Wang et al. analyzed BRCA1 expression in 418 patients who underwent surgical resection of NSCLC, followed by platinum-based therapy, and found no significant association between BRCA1 expression and response to treatment or survival [49]. In another subset of NSCLC, an in vitro analysis of cisplatin-resistant KRAS^G12C^ expressing tumors found an increased level of base excision repair proteins, which was hypothesized to confer resistance to cisplatin through the rapid removal of platinum-DNA adducts prior to crosslinking [50]. Moreover, an analysis of individual polymorphisms of four genes in lung tumors within the base excision repair pathway found that those that reduced DNA damage repair were associated with greater response to platinum-based chemotherapy [51]. Regarding genomic DDR alterations, Dai et al. found no predictive role of DDR deleterious alterations and survival in NSCLC treated with chemotherapy [52]. Patients who had alterations in base excision repair and nucleotide excision repair pathways had worse PFS in LUAD and LUSC, respectively. Conversely, another group found an association between shorter OS and PFS in patients with homologous recombination pathway alterations who had LUSC and were treated with carboplatin and nab-paclitaxel [53]. Therefore, the clinical utility of DDR alterations or protein expression to predict chemosensitivity and survival has not been supported in the literature to date.

Mechanisms of NSCLC radioresistance have recently been elucidated, implicating DDR pathway activation. Several pre-clinical studies utilizing siRNA knockdown techniques have identified important mediators. *RAD50*, a gene-encoding double-strand break-repair protein, was found to confer resistance to radiotherapy in a model of NSCLC [54]. The authors demonstrated that RAD50 over-expression was correlated with shorter disease-free survival and radioresistance in lung cancer patients who underwent tumor resection followed by post-operative radiation therapy [54]. Additionally, DNA damage binding protein 2 (DDB2) was shown to promote resistance through its role in DDR enhancement of the HR pathway, as well as playing a role in NER [55,56].

The advent of the immune checkpoint blockade represented a major advancement in the treatment of NSCLC. The search for predictive biomarkers for immunotherapy response is ongoing, as only a subset of cancer patients show objective responses with durable remission. Given the association between neoantigen expression and response to immunotherapy, it has been proposed that mutations in DDR pathways could be associated with improved ICI outcomes [57]. Recent studies show an association between mutation in several DDR pathways (including homologous recombination, base excision repair, nucleotide excision repair, and non-homologous end joining) and increased tumor mutational burden (TMB) in NSCLC [52,58,59]. Ricciuiti et al., demonstrated that pathogenic DDR mutations (shown in Figure 2, Panel C) were associated with improved response rate, PFS, and OS in NSCLC patients treated with PD-(L)1 inhibitors as a monotherapy or in combination with a CTLA-4 inhibitor [33]. Among 266 patients, those who had deleterious alterations in DDR genes had higher objective response rates (ORR) of 30.3% versus 17.2% in patients without identified pathogenic DDR mutations (*p* = 0.01). The median PFS and OS were longer in DDR-altered patients compared to patients without DDR alterations (median PFS 5.4 vs. 2.2 months, median OS 18.8 vs. 9.9 months, respectively, and both statistically significant). The survival benefit was also replicated when adjusting for TMB and PD-L1 expression. However, the predictive role of DDR alterations was incompletely answered in this study regarding patients who received chemoimmunotherapy, as those patients were not included in the study. Similarly, another study demonstrated that the presence of co-mutations in the DDR pathway (including *TP53*) served as a predictor of response to atezolizumab in NSCLC, even in patients with negative or low PDL-1 expression [60]. The authors defined co-mutation as the presence of DDR alterations in two or more DDR pathways (including 29 DDR genes from HR, MMR, BER, NER and NHEJ).

Finally, the study of the role of HRD score as a biomarker of response to different therapeutics in NSCLC is still under investigation. Doissy et al. demonstrated an exceptional duration of response of more than 20 months in a LUSC patient who received platinum-based therapy and had a high HRD score which ranked amongst the highest of 489 lung cancer cases in the GDC data portal. Moreover, the authors suggested that a high HRD score (>0.70), using HRDetect assay, was predictive of lung cancer cell line sensitivity to olaparib and talazoparib, suggesting a possible role of PARP inhibitors in a select population [46].

The results of these retrospective studies suggest that DDR alterations are not useful as predictive biomarkers for chemosensitivity but may have a role in predicting radio-resistance and response to immunotherapy in lung cancer. The integration of such biomarkers in the clinical setting (especially in the case of DDR alterations and immunotherapy) will depend on further validation in large prospective cohorts in NSCLC patients.

## 4. The Clinical Relevance of Targeting HR Pathway in Patients with HRD in NSCLC

Most of the data on the clinical efficacy of targeting HRD cancers come from PARPi trials in ovarian, breast, prostate, and pancreatic cancers. These tumors harbor HR deficiencies either due to *BRCA1/2* mutations or other HR-DDR alterations that render malignant cells susceptible to the inhibition of both BER and NHEJ pathways by trapping the PARP1 enzyme. Several PARPi have been approved by the FDA and EMA including olaparib, talazoparib, niraparib, and rucaparib. Initial preclinical studies showed that *BRCA-2-*deficient cell lines were sensitive to PARP inhibitors, human breast cancer cell lines, and tumor xenografts. Both PARP-1 and PARP-2 were inhibited; however, the reversal of the homologous recombination activity eliminated sensitivity [61]. In another key preclinical study, the combination of AZD2281 (olaparib, LYNPARZA™, AstraZeneca) and a platinum drug (cisplatin or carboplatin) in a genetically engineered mouse model with mammary tumors demonstrated improved recurrence-free survival and OS [62]. In vitro, olaparib-induced cytotoxicity has been shown to involve a reduction in PARP enzymatic activity and enhanced formation of the PARP-DNA complex, resulting in cell homeostasis disruption and cell death in NCI-60 cell lines [63]. Several phase-3 randomized controlled trials demonstrated improved PFS and OS using different PARPi in *BRCA1/2* mutated breast and ovarian cancers. Interestingly, the benefit was also noted in some wild-type *BRCA* tumors, which was later shown to be mediated by other deleterious deficiencies in DDR pathway, leading to an HRD state. This was demonstrated in a phase-3 randomized trial (PAOLA1) for the maintenance of olaparib and bevacizumab in a first-line setting in HRD-positive ovarian cancer, which improved PFS, and another randomized phase-3 trial in metastatic castration-resistant prostate cancer with alterations in HR genes (PROfound study), which demonstrated improved PFS [64,65]. These encouraging results led to several efforts to test the efficacy of these medications in other tumor types, including lung cancer.

Several trials have been conducted on the efficacy of PARPi in NSCLC given their sensitivity to platinum-based regimens, which is a predictive factor for response to PARP inhibition [66,67]. However, these trials did not demonstrate improved outcomes with the use of PARPi. The PIN trial, a phase-2 randomized-control trial, assigned 70 platinum-sensitive NSCLC patients, irrespective of BRCA mutation status, to either olaparib or placebo. Patients receiving olaparib had a notably longer, though not statistically significant, PFS (16.6 months as compared to 12 months, HR 0.83, 80% CI 0.6–1.15, *p* = 0.23). Of note, these patients were not required to have documented DDR pathway mutations [68]. S1900A, a phase-2 Lung-MAP sub-study, investigated whether NSCLC patients with demonstrated HRD had improved outcomes with rucaparib. They enrolled NSCLC patients with a documented high genomic loss of heterozygosity (LOH) or known *BRCA1/2* mutations who had disease progression on standard platinum-based or anti-PD-(L)1 therapy. The study was closed after interim analysis demonstrated futility with insufficient responses, however, 3 out of 4 total responders to rucaparib demonstrated *BRCA1* or *BRCA2* mutations [69]. This suggests that NSCLC patients with *BRCA1/2* mutations may have non-BRCA related alterations, leading to patients’ benefitting from the addition of PARPi therapy. Further supporting this, a 26-year-old woman with stage 4 adenocarcinoma found to have a germline mutation in BRCA2 (S497*) without other targetable mutations had an excellent clinical response to combination pembrolizumab and olaparib [70]. Importantly, preclinical data have offered evidence that olaparib upregulates PD-L1 expression, suggesting potential therapeutic synergy with checkpoint blockade [71]. Currently, the KEYLYNK-006 study (NCT03976323) is investigating the efficacy of the combination pembrolizumab with maintenance olaparib vs. maintenance pemetrexed in non-squamous NSCLC; however, patients are not required to have mutations in the DDR pathway.

Discoveries of targetable driver mutations have led to a significant improvement in the prognosis and treatment of NSCLC. However, this has largely only benefited patients with adenocarcinoma, a histologic subset of NSCLC. Squamous cell Carcinoma (LUSC), which represents approximately 30% of all NSCLC, has not had the same success in the identification and development of targeted therapies for driver mutations [72]. Given the high rate of high tumor mutation burdens in LUSC, several studies have been conducted to address the role of PARPi in these tumors. S1400AG, a Lung-MAP sub-study, evaluated the efficacy of PARPi talazoparib in HRD-LUSC [73]. The primary analysis population were required to have mutations in the following genes: *ATM, ATR, BRCA1, BRCA2,* and *PALB2*; however, the full eligible population of the study included mutations in additional HR pathway genes, including the Fanconi Anemia (FA) repair pathway. A total of 47 patients were enrolled, with 24 included in the primary analysis population. Overall, the study failed to demonstrate the efficacy of talazoparib. Interestingly however, 4 of 5 patients with objective response had alterations in the *FANCM*, *FANCC*, or *CHEK1* genes, which were not included in the primary analysis population. Therefore, further investigation into the potential therapeutic benefit of using PARPi in LUSC patients with FA repair pathway mutations may be warranted.

A recent large phase-3 randomized multicenter trial compared the use of veliparib in addition to platinum-based chemotherapy in patients with advanced squamous cell lung cancer in the first-line setting versus the use of chemotherapy alone. The rational of PARPi use in the first-line setting was due to the fact that DNA damage plays an important role in LUSC and due to preclinical studies demonstrating the efficacy of PARPi in LUSC with deficient DDR [74,75], and prior clinical evidence from a prior randomized phase-2 trial showing the benefits of PARP inhibition in squamous NSCLC [76]. The confirmatory phase 3 trial failed to demonstrate improved OS with the addition of veliparib with comparable safety [77]. Authors performed correlative biomarker analysis of the impact of binary gene-expression tumor-profiling measured by qRT-PCR (referred to as LP52) [78]. The authors studied the molecular features of squamous cell lung cancer and, based on gene expression, was classified as LP52-positive (molecular expression consistent with non-adenocarcinoma histology) and LP52-negative (adenocarcinoma molecular features). The authors found that LP52-positive patients had improved OS with the addition of veliparib and LP52-negative patients had a better outcome with chemotherapy alone (14 months vs. 6 months), suggesting that selected patient populations may benefit from the addition of PARPi; however, DDR alterations were not included in gene expression profiling. Another phase-3 randomized clinical trial in advanced non-squamous NSCLC failed to demonstrate a benefit of adding veliparib to chemotherapy backbone in first-line treatment, although OS was numerically higher (11.2 months vs. 9.2 months, respectively, *p* = 0.113) [79].

Despite the lack of efficacy of PARPi with chemotherapy in several lung cancer trials, there is an ongoing effort to investigate the possible role of combining PARPi with immunotherapy in NSCLC due to the synergistic effect of the combination treatment and acceptable safety [80,81]. The GUIDE2REPAIR trial (NCT04169841) is an ongoing, Phase-2, non-randomized study, which seeks to assess the safety and efficacy of combination olaparib, durvalumab and tremelimumab in patients with metastatic solid-tumor malignancy, including NSCLC, and the identified mutations in HR pathway genes [82]. The UNITO-001 (NCT04940637) is another prospective, single-arm, phase-2 trial investigating the safety and efficacy of combination niraparib and dostarlimab in patients with advanced NSCLC and malignant pleural mesothelioma with germline or somatic mutations in HR genes and positive PD-L1 expression (TPS ≥ 1%). At present, they are actively enrolling patients, and have an estimated completion date of January 2024 [83]. Table 1 summarizes the current PARPi that are in clinical trials in NSCLC [84,85,86,87]. Recently, Marzio et al. demonstrated the role of Klech-like ECH-associated protein 1 (*KEAP1)* mutation, which is an important redox hemostasis regulator, in producing BRCAness phenotype in NSCLC [88]. The investigators demonstrated that KEAP1 knockout led to the stabilization of EMSY, an important inhibitor of BRCA2, which led to a homologues’ recombination-deficient state. In addition, accumulation of EMSY was associated with type I interferon suppression, which, in turn, promoted immune escape. The administration of PARP inhibitors had anti-tumor efficacy in KEAP1 mutant NSCLC and this was further augmented with the addition of STING agonist, providing a rationale for the use of PARP inhibitors in NSCLC with BRCAness phenotype due to the KEAP1 mutation.

Beyond PARP inhibition, most of the other novel DDR inhibitors are currently being studied in early-phase clinical trials in solid tumors. Preclinical evidence suggests that the inhibition of ATR in NSCLC cell lines increases sensitivity to both cisplatin and radiation therapy [89,90]. A phase 1b/2 trial is underway to evaluate the safety and efficacy of adding an ATR inhibitor berzosertib (M6620 VX-970) to chemotherapy and immunotherapy in metastatic squamous NSCLC (NCT04216316). Another phase 1 trial is evaluating the safety of the addition of berzosertib to whole-brain radiation for the treatment of brain metastases of SCLC and NSCLC (NCT02589522). Another ATR inhibitor, ceralasertib (AZD6738), is currently being evaluated in a phase 2 umbrella trial in NSCLC (the HUDSON trial) in patients who progressed on anti-PD-(L)1 therapy (NCT03334617). AZD6738, which is an ATM/ATR inhibitor, has demonstrated acceptable safety in phase 1 clinical trials in solid tumors when combined with carboplatin, with olaparib or with durvalumab [91]. Similarly, other inhibitors of DDR proteins, such as ATM, CHEK1/2, WEE1 and DNA-PK, are currently being evaluated in early-phase clinical trials in solid tumors including NSCLC, and are summarized in Supplementary Table S1.

In summary, PARP inhibitors did not demonstrate clinical efficacy in NSCLC despite the notion that platinum-sensitive cancers could be responsive to PARPi. Further studies are needed to analyze the efficacy of PARPi in the subset of patients with specific DDR alterations that could lead to *BRCA* deficiency or an HRD phenotype. This will require further understanding of the biological implications of specific DDR alterations on cancer cell dependence on compensatory repair pathways. The development of validated HRD assays specific to lung cancer could be of interest regarding whether they could select for a biologically distinct tumor subtype that could be more sensitive to DDR targeting the containing regimen. Furthermore, the combination of PARPi with checkpoint inhibitors is of interest, given the preclinical evidence of synergism and relative safety compared to combining PARPi with chemotherapy.

## 5. Prognostic and Predictive Biomarkers in DNA Damage Pathway Alterations in SCLC

Tumor-suppressor genes of retinoblastoma protein (RB) and the p53 transcription factor are one of the most commonly mutated genes across all cancer types. SCLC harbors *RB* loss-of-function alterations in approximately 80% of cases, and *TP53* is mutated in the majority of SCLC [92]. Alterations in the DDR genes are infrequently present in SCLC (with the exception of *TP53*), as discussed in Section 2. However, the overexpression of several DDR-related proteins, such as PARP1, has been linked to the pathogenesis and response to treatment. In SCLC, cell line showed the highest PARP1 expression of any solid tumor line, including breast and ovarian cells, among individual cell lines [37]. *PARP1* and *EZH2* gene knockdowns decreased SCLC growth. When compared to NSCLC, SCLC is much more responsive to PARP inhibitors, and PARP inhibition downregulated critical components of the DNA repair mechanism while improving chemotherapy effectiveness in SCLC [37].

There have been several attempts to use HRD assays as a predictive tool of response to PARPi in SCLC. Those assays, based on an analysis of preexisting genomic mutations, did not show correlations with SCLC sensitivity to PARP inhibition. However, it was found that schlafen family member 11 (SLFN11) correlated with PARPi sensitivity and could be used through CRISPR/Cas9 and shRNA approaches to knock out SLFN11 [93]. Several studies showed different SLFN11 results as a predictive marker, as some studies supported that high expression of SLFN11 correlated with response to PARP inhibitors. In the Lok et al. study, it was shown that SLFN11 was a relevant predictive biomarker of susceptibility to PARP inhibitor monotherapy in SCLC. Combination treatment with temozolomide (TMZ) stood out as a particularly interesting therapeutic strategy. Patients with SLFN11-positive tumors treated with TMZ and veliparib showed prolonged PFS (5.7 vs. 3.6 months; *p* = 0.009) and OS (12.2 vs. 7.5 months; *p* = 0.014) [93]. Similarly, Pietanza et al. found a considerable improvement in ORR with TMZ and veliparib in patients receiving this combination, where SLFN11 expression in tumors was related to better PFS and OS, indicating a predictive biomarker of PARP-inhibitor sensitivity in SCLC [94]. Murai et al. investigated SLFN11 as a potentially relevant biomarker of response for patients treated with PARPi, as well as for combining ATR and PARP inhibitors to overcome SLFN11-negative cells’ resistance to PARPi. They identified SLFN11 as a causative and dominating predictor of cellular responsiveness to PARPi (talazoparib and olaparib), both alone and in combination with TMZ. The study showed that SLFN11 affects cellular responses to PARPi independently of homologous recombination and drug efflux by inhibiting DNA replication without the involvement of ATR [63]. Gay et al. defined four subgroups of SCLC, each with distinct molecular characteristics and treatment vulnerabilities. The clinical implications of this subtype categorization are considerable, as each subtype is vulnerable to experimental medicines in a distinct way. For example, for PARP inhibitors, strong SLFN11 expression is the dominant predictive biomarker at present [95].

Cardnell et al. investigated the action of BMN 673, a novel, PARP inhibitor. In SCLC, the DNA Repair score (including genes such as *BRCA*, *ATM*, *ATR*, *CHK*, *RAD50* and *FANC*) was shown to correlate with the response of PARPi. The DNA repair score has a high inverse correlation to BMN 673 IC50, which is consistent with the expression of specific DNA repair markers (ρ = −0.762, *P* = 0.037), corroborating the result that cell lines with the greatest overall expression of DNA repair makers are the most susceptible to BMN 673 [96]. Finally, common alterations in SCLC, such as MYC amplification, have been shown to correlate with response to DNA-targeting agents such as CHEK1 inhibitors and in combination with cisplatin and olaparib, providing a rationale that subtypes of SCLC could be more vulnerable to DDR inhibition [97]. Therefore, no specific DDR-related alterations have been found to be predictive of treatment response in SCLC. This could be due to the scarcity of DDR-related alterations in small cell lung cancer, as well as their minor biological role. Nevertheless, non-DDR related biomarkers, such as SLFN11 and MYC amplification, are likely to be selected for further studies to assess their role in predicting response to different SCLC treatments, including PARPi.

## 6. Clinical Relevance of Targeting HR Pathway in Patients with HRD in SCLC

PARP inhibitors have been widely studied in SCLC. A phase 1, dose-escalation, two-part trial evaluated the efficacy of the PARP inhibitor talazoparib (MDV3800, BMN 673, talzenna, Pfizer). The study enrolled 23 patients with SCLC who were treated with 1 mg/day of talazoparib. Only two patients showed partial response and four patients had stable disease (SD) of at least 16 weeks [98]. In a phase-2 STOMP trial, olaparib was studied as a maintenance treatment for 220 patients with chemo-responsive SCLC. Two regimens of olaparib were investigated: 300 mg twice daily and 200 mg three times daily. There was no significant difference in PFS or OS between placebo and both treatment groups [99]. Veliparib was also studied in combination with platinum-based drugs. A phase 1 dose-escalation study evaluated the combination of veliparib with carboplatin and etoposide. In 25 patients with extensive-stage SCLC, complete responses (CR) and partial responses (PR) were observed in 64% of patients [100]. In a phase 2 trial that explored the efficacy of cisplatin and etoposide in combination with veliparib or placebo in extensive-stage SCLC. A total of 128 patients were enrolled in the study. The addition of veliparib to the regimen moderately improved outcomes for the patients with the median PFS for the veliparib arm versus the placebo arm of 6.1 versus 5.5 months. The median OS was 10.3 versus 8.9 months [101]. In another study, patients with relapsed SCLC in TMZ and veliparib group showed no signficiant difference to the TMZ and placebo group in 4-month PFS and OS. However, ORR was significantly better in TMZ and veliparib group. As mentioned earlier, SLFN11 expression was correlated to the efficacy of TMZ and veliparib showing improved PFS and OS [94]. Olaparib was also investigated in the combination with TMZ in a phase 1/2 study. The ORR was 41.7%, median PFS was 4.2 and median OS was 8.5 months [102]. PARPi (veliparib and talazoparib) were also shown to radiosensitize SCLC cell lines, and talazoparib was proven to be superior in this regard, providing a rationale for the design of trials combining PARPi with radiation therapy [103].

PARPi are currently also being investigated in combination with targeted therapy [104]. Checkpoint kinase 1 (CHK1) inhibitor prexasertib (LY2606368) increased the efficacy of olaparib in cell lines as well as xenograft models. This combination decreased viability and caused tumor regression [97]. Olaparib was also studied in combination with the WEE1 G2 checkpoint kinase inhibitor AZD1775. In a xenograft model, this combination was shown to be superior to the standard therapy of cisplatin/etoposide [105]. In Murai, Junko et al.’s study in different cell lines, SLFN11 expression was inversely correlated with sensitivity to PARP-trapping inhibitors (olaparib and talazoparib). Therefore, the increase in SLFN11 expression with the use of ATR inhibitor (VE-821) was suggested to lead to the higher sensitivity to PARPi [63]. The use of EPZ011989 (EZH2 inhibitor) in cell lines also increased SLFN11 expression and could potentially confer higher sensitivity to PARPi [106].

Other PARPi combinations with different therapeutics are currently being studied. For example, there is an ongoing phase 2 study of cediranib in combination with olaparib in multiple cancer types including SCLC (NCT02498613) [107]. This follows the evidence from other tumor types that combine olaparib and cediranib, which is a vascular growth epidermal factor (VGEF) inhibitor that has shown improved PFS and OS [108]. Moreover, cediranib was found in breast and ovarian cancer xenografts to suppress the expression of BRCA1/2 and RAD51, inducing HRD state [109]. Another combination with PI3K inhibitors has been proposed by Cardnell et al., who showed, using a reverse-phase protein array, that PI3K/mTOR pathway (p-mTOR, pAKT, and pS6) is highly upregulated following the PARPi treatment. They hypothesize that this could be due to decreased ATP usage and a decrease in stress response signaling via liver kinase B1 (LKB1). Thus, they proposed combining talazoparib and BKM-120, and the effect was proven in vitro [110].

Immunotherapy shows a durable response in only a small fraction of patients with SCLC. This response could be defined by immune tumor subtype. Therefore, using a combination therapy that could reprogram the tumor microenvironment to be more sensitive to immunotherapy represents a possible avenue to be explored. PARPi have been described to increase the accumulation of cytosolic DNA fragments that activated the DNA-sensing cGAS–STING pathway and stimulated the production of type-1 IFNs. This, in turn, stimulates antitumor immunity and-infiltrating lymphocytes, which could be further amplified using checkpoint inhibitors [111]. A study showed that PARPi upregulated PD-L1 expression in breast cancer cell lines and animal models, which led to higher anticancer immunity. It was also shown that the combination of PARPi and anti-PD-L1 therapy had a significantly increased therapeutic efficacy in vivo [71]. PARPi were studied in combination with durvalumab. Patients with recurrent SCLC were included in a single-arm, phase 2 study (NCT02484404) and were given durvalumab, 1500 mg, every 4 weeks, and olaparib, 300 mg, twice a day. However, the trial combination did not reach the effectiveness threshold. Biopsy specimens taken before and during therapy revealed that tumor immunological phenotypes may be important for SCLC responses to immune checkpoint blockade combinations [112]. At present, rucaparib is being investigated in combination with anti-PD-1 drug nivolumab in several phase 1/2/3 studies in different cancer types, including SCLC [113]. Finally, talazoparib is currently being tested in a phase 2 rial in combination with atezolizumab, to determine if this combination would be superior to atezolizumab maintenance in patients with extensive SCLC who have SLFN11-positive tumors (NCT04334941).

A major potential limiting factor for the combination of PARPi with immunotherapy is its safety profile, as the majority of patients could develop side effects, with about one third of patients possibly experiencing grade 3/4 side effects [114]. Therefore, further studies are essential to investigate the safety profile of combination PARPi and immunotherapy, especially given that different PARPi have distinct potency and overlapping side effects with checkpoint blockade (such as hepatitis).

## 7. Conclusions

The DNA-Damage Repair (DDR) pathways consists of multiple genes that are involved in maintaining genomic stability. Disruption in DDR pathways can lead to cancer progression, and provides the rationale for exploiting repair deficiencies through therapeutic intervention such as in breast and ovarian cancers. The role of DDR in lung cancer is unclear and requires further investigation due to the prevalence of DDR-related alterations in lung cancer (specifically in NSCLC). Further validation is needed to confirm DDR pathway alterations as a predictive marker of immunotherapy response in NSCLC. However, if confirmed, this will be an extremely important marker to consider when selecting patients for clinical trials and potential PARPi combination therapy with immunotherapy. In contrast, DDR alterations appear to be less reliable as a predictive biomarker of response to chemotherapy or radiation therapy in NSCLC. In SCLC, the available evidence suggests no apparent role of DDR alterations as predictors of response to treatment; however, the presence of surrogate non-DDR-related markers (such as SLFN11) could help select for patients responsive to PARP inhibition.

More focused research efforts are essential to better understand the biological implications of DDR alterations in both NSCLC and SCLC, as there has been no demonstration of the efficacy of DDR-targeting agents, despite several clinical trials. Understanding how DDR pathway alterations and treatments remodel the tumor microenvironment will provider future opportunities to study additional novel therapeutic strategies that may impact outcomes in patients with lung cancer who harbor DDR-pathway alterations.

## Figures and Tables

**Table 1:** A summary of selected clinical trials with PARPi in NSCLC.

Clinical Trial	Medication	Tumor Type	Inclusion Criteria	Exclusion Criteria	Selective Biomarkers	End Point	Results	Ref.
NCT01513174	Olaparib + Gefitinb vs. Gefitinib alone Phase 1 and Phase 2	NSCLC	Stage 4 disease ECOG score ≤ 2. Life expectancy of ≥ 3 months.	Prior treatment with PARPi History of ILD 2nd neoplasm	EGFR+	Primary: Maximum tolerated dose Secondary: PFS, ORR, OS	mPFS 12.8 vs. 10.9 (*p* = 0.124) mOS 23.3 vs. 23.1 (*p* = 0.345)	[84]
NCT01562210	Olaparib dose escalating + RT with or without cisplatin Phase 1	NSCLC	Stage 2/3 non-operable disease Life expectancy of ≥ 3 months	Prior radiation/chemotherapy Significant pulmonary, cardiovascular, hepatic disease	Targetable mutations	Primary: dose-limiting toxicity Secondary: PFS, ORR	Dose limiting toxicity due to esophageal, hematologic and pulmonary toxicity	[85]
NCT03775486	Durvalumab + Olaparib vs Durvalumab Phase 2	NSCLC	Stage 4 NSCLC, not amenable to curative surgery or radiation	History of autoimmune disease Prior chemo/immune therapy	EGFR mutation negative ALK fusions negative	Primary: PFS Secondary: OS, ORR	N/A	Ongoing trial
NCT03976362	Pembrolizumab +/- maintenance Olaparib Phase 3	squamous NSCLC	Stage 4, life expectancy ≥ 3 months	History of autoimmune disease History of ILD	N/A	Primary: PFS, OS Secondary: AE, QLQ-C30	N/A	Ongoing trial
NCT04380636	Pembrolizumab with chemoradiation, followed by pembrolizumab +/− Olaparib Phase 3	NSCLC	Stage 3, unresectable, life expectancy ≥ 6 months	Prior treatment History of autoimmune disease	N/A	Primary: PFS, OS Secondary: AE, ORR, DOR, QLQ-C30	N/A	Ongoing trial
NCT03976323	Pembrolizumab followed by maintenance Olaparib vs. pemetrexed Phase 3	Non-squamous NSCLC	Stage 4, life expectancy ≥ 3 months	History of autoimmune disease Prior therapy with PARPi CNS disease	EGFR, ROS1 mutations negative, ALK fusions negative	Primary: PFS, OS Secondary: AE, ORR, QLQ-C30	N/A	Ongoing trial
NCT03334617	Umbrella study of novel agents (Olaparib) +Durvalumab Phase 2	NSCLC	Stage 4, progressed on anti PD1/PD-L1, life expectancy ≥ 3 months	History of primary immunodeficiency or autoimmune disorders, CNS disease	EGFR mutation negative ALK fusions negative ROS1, BRAF, MET, RET negative	Primary: ORR Secondary: DCR, DOR, PFS, OS	N/A	Ongoing trial
NCT04550104	Platform study of novel agents (olaparib) with radiotherapy Phase 1B	NSCLC	Stage 2B/3A/3B, not eligible for concurrent chemoradiotherapy/surgery	Prior autoimmune or inflammatory disorders, history of interstitial pneumonitis	N/A	Primary: Dose-limiting toxicities Secondary: ORR, OS, PFS, QLQ-C30	N/A	Ongoing trial
NCT03559049	Rucaparib and Pembrolizumab for maintenance therapy Phase 1/2	Non-squamous NSCLC	Stage 4, life expectancy ≥ 3 months	Prior systemic therapy, prior treatment with PARPi, history of autoimmune disease	EGFR mutation negative ALK fusions negative ROS1 negative	Primary: PFS Secondary: OS, ORR	N/A	Ongoing trial
NCT03845296	Rucaparib (LUNG-MAP Sub-Study) Phase 2	NSCLC	Stage 4	Prior treatment with PARPi, Grade 3/4 CV disease,	Genomic LOH or BRCA 1, 2 mutations ALK fusions negative EGFR, ROS1, BRAF mutations negative	Primary: ORR Secondary: IA-PFS, OS, DOS	N/A	N/A
NCT03891615	Phase 1—Niraparib with combination osimertinib in EGFR mutated lung cancer	NSCLC	Stage 4, EGFR mutant NSCLC, life expectancy > 6 months	No prior PARP inhibitor, prior immunotherapy, ILD,	EGFR	Primary: Max tolerated dose Secondary: Rate of toxicity in combined, ORR niraparib, median PFS niraparid	N/A	Ongoing trial
NCT03497429	Phase 1 niraparib in Japanese pts w/advanced solid tumors	Adv Solid tumor	Met or locally advanced solid tumor malignancy, Japanese, >20 years old	Prior PARPi, CNS mets,	N/A	Primary: Dose-limiting toxicity Secondary: Plasma concentration, AUC	N/A	N/A
NCT05169437	Phase 2 niraparib in locally advanced on met PALB2 mutations	Adv Solid Tumor	Met or locally advanced, Pathogenic PALB2	Ovarian or prostate ca, prior ParpI therapy, BRCA1 or 2,	Pathogenic PALB2 +, BRCA 1 and BRCA 2 negative	Primary: ORR Secondary: PFS, ORR, AE events, OS	N/A	Ongoing trial
NCT04475939	ZEAL-1L—Phase 3 RCT, Niraparib + pembro vs. Placebo + pembro in NSCLC	NSCLC	Met or locally advanced, life expectancy > 12 wks	Prior ParpI, brain mets, autoimmune disorder,	N/A	Primary: PFS, OS Secondary: TTP, PFS by PDL1 status, IRAEs	Results from phase 2 trial: ORR 56.3% with TPS ≥ 50 (Cohort 1) and 20% in TPS 1–49% (Cohort 2). mPFS: 8.4 in cohort 1 and 4.2 in cohort 2. mOS NR in cohort 1 and 7.7 in cohort 2	[86]
NCT03308942	Phase 2 niraparib + anti -PD-1 (Pembro vs. Dostarlimab)	NSCLC (cohort 3—sqaumous)	Met or locally advanced, Cohort 1—PDL-1 > 50% cohort 2- PDL-1 1–49%; Cohort 3—SqCC,	Cohort 1/2; Prior Anti-PD1, PDL-1 or PDl-2 therapy All cohorts- hx of immunodeficiency, hx of autoimmune disease, ILD or pneumonitis	ROS-, ALK-, EGFR-	Primary: ORR Secondary: Non-SAEs, SAEs, duration of response, PFS	N/A	N/A
NCT03209401	Phase 1—Niraparib + carboplatin in HRR deficient adv solid tumors	Adv solid tumor	Life expectancy > 3 mo,	Prior platinum chemotherapy w/in last 6 mo. Prior PARPi, CNS disease,	HR pathway mutations	Primary: Grade 3–4 toxicities, ORR Secondary: Median survival, PFS, DCR	N/A	N/A
NCT02944396	Veliparib in combination with Nivolumab and Platinum doublet Phase 1	NSCLC	Stage 4 or unresectable Stage 3B Life expectancy > 3 months	Prior therapy with PARPi or checkpoint inhibitor CNS disease	N/A	Primary: PFS, RPTD Secondary: OS, AUC, DOR, Cmax	N/A	N/A
NCT02264990	Veliparib + carboplatin and paclitaxel v investigator’s choice chemotherapy Phase 3	Non-squamous NSCLC	Stage 3 unresectable, stage 4 Current or former smoker	Peripheral neuropathy, Prior cytotoxic chemo or radiation for NSCLC	EGFR mutation negative ALK fusion negative LP52+	Primary: Number of participants with DLT Secondary: ORR	mOS 12.1 in both arms (*p* = 0.846)	[79]
NCT01560104	Veliparib vs. placebo in combination with carboplatin and paclitaxel Phase 2	NSCLC	Stage 3 unresectable, stage 4 Life expectancy > 3 months	Peripheral neuropathy, Prior cytotoxic chemo or radiation for NSCLC	EGFR mutation negative	Primary: PFS Secondary: OS, ORR, CIPN	mPFS 5.8 months vs. 4.2 months (*p* = 0.17). mOS 11.7 vs. 9.1 (*p* = 0.27). ORR: 32.4% vs. 32.1%	[76]
NCT012106546	Veliparib vs. placebo in combination with carboplatin and paclitaxel Phase 3	Squamous NSCLC	Stage 3 unresectable, stage 4 Life expectancy > 3 months	Peripheral neuropathy, Prior cytotoxic chemo or radiation for NSCLC	EGFR mutation negative ALK fusion negative	Primary: OS (current smokers) Secondary: OS (all subjects), PFS, ORR	mOS 11.9 months vs. 11.1 months (*p* = 0.266).	[77]
NCT01657799	Veliparib 50 BID with WBRT vs. veliparib 200 BID with WBRT vs. placebo with WBRT for NSCLC Brain metastases Phase 2	NSCLC	Brain mets on MRI, eligible for WBRT, adequate hematologic, renal, and hepatic function	Dx of brain mets > 28 days prior to day 1, more than 2 sites of metastasis, leptomeningeal spread, prior PARPi therapy	N/A	Primary: OS Secondary: Best tumor response rate, time to intracranial progression	mOS 209 days vs. 209 days vs 185 days *p* >0.05	[87]
NCT01386385	Veliparib with or without radiation, carboplatin, and paclitaxel Phase 1 followed by Phase 2	NSCLC	Stage 3 unresectable disease	Prior cytotoxic chemo or radiation for NSCLC Immune disorder	N/A	Primary: Maximum tolerated dose, PFS Secondary: OS, ORR, incidence of Grade 3+ AE	N/A	Ongoing
NCT01282333	Veliparib, cisplatin, and gemcitabine hydrochloride Phase 1	NSCLC, (+biliary, pancreatic, urothelial cancer	Stage 3 unresectable, Stage 4	Prior cytotoxic chemo or radiation	BRCA1 or BRCA 2 germline mutation	Primary: Maximum tolerated dose Secondary: Dose-limiting toxicities, antitumor activity	N/A	N/A (terminated)
NCT04173507	Talazoparib plus avelumab (LUNG-MAP trial) Phase 2	NSCLC	Stage 4 or recurrent disease Prior treatment with one line of anti-PD-1 or PD-L1	Prior PARPi, Grade 2/4 cardiac disease,	STK11 gene mutation	Primary: ORR Disease control rate Secondary: Toxicities, PFS, OS, Duration of response	N/A	Ongoing trial
NCT03330405	Avelumab plus Talazoparib Phase 1/2	NSCLC, Ovarian, urothelial, TNBC	Stage 3 unresectable, Stage 4, ECOG 1, 2	Prior PARPi, history of brain mets requiring steroid, autoimmune disease	BRCA, ATM mutations	Primary: DLT, OR Secondary: TTR, DOR, PFS, OS	N/A	Ongoing trial

Abbreviations: NSCLC: non-small cell lung cancer; EGFR: epidermal growth factor receptor; ILD: interstitial lung disease; RT: radiation therapy; PFS: progression-free survival; OS: overall survival; N/A: not available; AE: adverse events; QLQ-C3-: Quality of Life Questionnaire-Core 30; ORR: objective response rate; DOR: duration of response; DCR: disease control rate; IA-PFS: investigator assessed progression-free survival; LOH: loss of heterozygosity; HR: homologous recombination; TTP: treatment to progression; IRAEs: immune-related adverse events; SAE: serious adverse events; AUC: area under curve; RPTD: recommended phase-2 dose; WBRT: whole-brain radiation therapy; TTR: time to tumor response.

## Supplementary Materials

The following supporting information can be downloaded at: https://www.mdpi.com/article/10.3390/cancers14215305/s1, Table S1: Select clinical trials of DNA-damage repair targeted agents in clinical trials in solid tumors including non-small cell lung cancer.

## Abbreviations

NSCLC: Non-small cell lung cancer; SCLC: small cell lung cancer; LAUD: lung adenocarcinoma; LUSC: lung squamous cell carcinoma; PD-L1: programmed death-ligand 1; Os: overall survival; ICI: immune checkpoint inhibitors; DDR: DNA-damage repair; TCGA: The Cancer Genome Atlas; NER: nucleotide excision repair; BER: base excision repair; DR: direct repair; HR: homologous recombination; NHEJ: non-homologous end joining; MMR: mismatch repair; GG-NER: global genomic nucleotide excision repair; TC-NER: transcription-coupled nucleotide excision repair; PARP: poly(ADP-ribose) polymerase; DSB: DNA double-strand breaks; HRD: homologous recombination deficiency; LOH: loss of heterozygosity; NtAI: number of telomeric allelic imbalances; LST: large-scale transitions; PARPi: PARP inhibitors; FA: Fanconi Anemia; PFS: progression-free survival; DDB2: DNA damage binding protein 2; TMB: tumor mutational burden; ORR: objective response rates; SLFN11: Schlafen family member 11; TMZ: temozolomide; SD: stable disease; VGEF: vascular growth epidermal factor.

## References

[1] Thai A.A., Solomon B.J., Sequist L.V., Gainor J.F., Heist R.S. (2021). Lung cancer. Lancet.

[2] Bray F., Ferlay J., Soerjomataram I., Siegel R.L., Torre L.A., Jemal A. (2018). Global cancer statistics 2018: GLOBOCAN estimates of incidence and mortality worldwide for 36 cancers in 185 countries. CA Cancer J. Clin..

[3] Skoulidis F., Heymach J.V. (2019). Co-occurring genomic alterations in non-small-cell lung cancer biology and therapy. Nat. Rev. Cancer.

[4] Imielinski M., Berger A.H., Hammerman P.S., Hernandez B., Pugh T.J., Hodis E., Cho J., Suh J., Capelletti M., Sivachenko A. (2012). Mapping the Hallmarks of Lung Adenocarcinoma with Massively Parallel Sequencing. Cell.

[5] Cancer Genome Atlas Research Network (2012). Comprehensive genomic characterization of squamous cell lung cancers. Nature.

[6] Skoulidis F., Li B.T., Dy G.K., Price T.J., Falchook G.S., Wolf J., Italiano A., Schuler M., Borghaei H., Barlesi F. (2021). Sotorasib for Lung Cancers with *KRAS* p.G12C Mutation. N. Engl. J. Med..

[7] Reck M., Rodríguez-Abreu D., Robinson A.G., Hui R., Csőszi T., Fülöp A., Gottfried M., Peled N., Tafreshi A., Cuffe S. (2016). Pembrolizumab versus Chemotherapy for PD-L1–Positive Non–Small-Cell Lung Cancer. N. Engl. J. Med..

[8] Felip E., Altorki N., Zhou C., Csőszi T., Vynnychenko I., Goloborodko O., Luft A., Akopov A., Martinez-Marti A., Kenmotsu H. (2021). IMpower010 Investigators. Adjuvant atezolizumab after adjuvant chemotherapy in resected stage IB–IIIA non-small-cell lung cancer (IMpower010): A randomised, multicentre, open-label, phase 3 trial. Lancet.

[9] Akinboro O., Vallejo J.J., Mishra-Kalyani P.S., Larkins E.A., Drezner N.L., Tang S., Pazdur R., Beaver J.A., Harpreet S. (2021). Outcomes of anti-PD-(L1) therapy in combination with chemotherapy versus immunotherapy (IO) alone for first-line (1L) treatment of advanced non-small cell lung cancer (NSCLC) with PD-L1 score 1–49%: FDA pooled analysis. J. Clin. Oncol..

[10] Knijnenburg T.A., Wang L., Zimmermann M.T., Chambwe N., Gao G.F., Cherniack A.D., Fan H., Shen H., Way G.P., Greene C.S. (2018). Genomic and Molecular Landscape of DNA Damage Repair Deficiency across The Cancer Genome Atlas. Cell Rep..

[11] Burgess J.T., Rose M., Boucher D., Plowman J., Molloy C., Fisher M., O’Leary C., Richard D.J., O’Byrne K.J., Bolderson E. (2020). The Therapeutic Potential of DNA Damage Repair Pathways and Genomic Stability in Lung Cancer. Front. Oncol..

[12] Melis J.P., van Steeg H., Luijten M. (2013). Oxidative DNA Damage and Nucleotide Excision Repair. Antioxid. Redox Signal..

[13] Beard W.A., Horton J.K., Prasad R., Wilson S.H. (2019). Eukaryotic Base Excision Repair: New Approaches Shine Light on Mechanism. Annu. Rev. Biochem..

[14] Takaoka M., Miki Y. (2017). BRCA1 gene: Function and deficiency. Int. J. Clin. Oncol..

[15] Hanahan D., Weinberg R.A. (2011). Hallmarks of cancer: The next generation. Cell.

[16] Pilié P.G., Tang C., Mills G.B., Yap T.A. (2019). State-of-the-art strategies for targeting the DNA damage response in cancer. Nat. Rev. Clin. Oncol..

[17] Nguyen L.W.M., Martens J., Van Hoeck A., Cuppen E. (2020). Pan-cancer landscape of homologous recombination deficiency. Nat. Commun..

[18] Abkevich V., Timms K.M., Hennessy B.T., Potter J., Carey M.S., Meyer L.A., Smith-McCune K., Broaddus R., Lu K.H., Chen J. (2012). Patterns of genomic loss of heterozygosity predict homologous recombination repair defects in epithelial ovarian cancer. Br. J. Cancer.

[19] Popova T., Manié E., Rieunier G., Caux-Moncoutier V., Tirapo C., Dubois T., Delattre O., Sigal-Zafrani B., Bollet M., Longy M. (2012). Ploidy and Large-Scale Genomic Instability Consistently Identify Basal-like Breast Carcinomas with *BRCA1/2* Inactivation. Cancer Res..

[20] Birkbak N.J., Wang Z.C., Kim J.-Y., Eklund A.C., Li Q., Tian R., Bowman-Colin C., Li Y., Greene-Colozzi A., Iglehart J.D. (2012). Telomeric Allelic Imbalance Indicates Defective DNA Repair and Sensitivity to DNA-Damaging Agents. Cancer Discov..

[21] Robson M., Im S.A., Senkus E., Xu B., Domchek S.M., Masuda N., Delaloge S., Li W., Tung N., Armstrong A. (2017). Olaparib for Metastatic Breast Cancer in Patients with a Germline BRCA Mutation. N. Engl. J. Med..

[22] Litton J., Hurvitz S., Mina L., Rugo H., Lee K.-H., Gonçalves A., Diab S., Woodward N., Goodwin A., Yerushalmi R. (2020). Talazoparib versus chemotherapy in patients with germline BRCA1/2-mutated HER2-negative advanced breast cancer: Final overall survival results from the EMBRACA trial. Ann. Oncol..

[23] Hussain M., Mateo J., Fizazi K., Saad F., Shore N., Sandhu S., Chi K.N., Sartor O., Agarwal N., Olmos D. (2020). Survival with Olaparib in Metastatic Castration-Resistant Prostate Cancer. N. Engl. J. Med..

[24] Yoshida K., Gowers K.H.C., Lee-Six H., Chandrasekharan D.P., Coorens T., Maughan E.F., Beal K., Menzies A., Millar F.R., Anderson E. (2020). Tobacco smoking and somatic mutations in human bronchial epithelium. Nature.

[25] Hopkin J.M., Evans H.J. (1980). Cigarette smoke-induced DNA damage and lung cancer risks. Nature.

[26] Zhao H., Albino A.P., Jorgensen E., Traganos F., Darzynkiewicz Z. (2009). DNA damage response induced by tobacco smoke in normal human bronchial epithelial and A549 pulmonary adenocarcinoma cells assessed by laser scanning cytometry. Cytom. Part A.

[27] Heeke A.L., Pishvaian M.J., Lynce F., Xiu J., Brody J.R., Chen W.-J., Baker T.M., Marshall J.L., Isaacs C. (2018). Prevalence of Homologous Recombination–Related Gene Mutations Across Multiple Cancer Types. JCO Precis. Oncol..

[28] Ding L., Getz G., Wheeler D.A., Mardis E.R., McLellan M.D., Cibulskis K., Sougnez C., Greulich H., Muzny D.M., Morgan M.B. (2008). Somatic mutations affect key pathways in lung adenocarcinoma. Nature.

[29] Parry E.M., Gable D.L., Stanley S.E., Khalil S.E., Antonescu V., Florea L., Armanios M. (2017). Germline Mutations in DNA Repair Genes in Lung Adenocarcinoma. J. Thorac. Oncol..

[30] Campbell J.D., Alexandrov A., Kim J., Wala J., Berger A.H., Pedamallu C.S., Shukla S.A., Guo G., Brooks A.N., Murray B.A. (2016). Distinct patterns of somatic genome alterations in lung adenocarcinomas and squamous cell carcinomas. Nat. Genet..

[31] Yoshino T., Pentheroudakis G., Mishima S., Overman M., Yeh K.-H., Baba E., Naito Y., Calvo F., Saxena A., Chen L.-T. (2020). JSCO—ESMO—ASCO—JSMO—TOS: International expert consensus recommendations for tumour-agnostic treatments in patients with solid tumours with microsatellite instability or NTRK fusions. Ann. Oncol..

[32] Waqar S.N., Devarakonda S.H.K., Michel L.S., Maggi L.B., Watson M., Guebert K., Carpenter D., Sleckman B.P., Govindan R., Morgensztern D. (2014). BRCAness in non-small cell lung cancer (NSCLC). J. Clin. Oncol..

[33] Ricciuti B., Recondo G., Spurr L.F., Li Y.Y., Lamberti G., Venkatraman D., Umeton R., Cherniack A.D., Nishino M., Sholl L.M. (2020). Impact of DNA Damage Response and Repair (DDR) Gene Mutations on Efficacy of PD-(L)1 Immune Checkpoint Inhibition in Non–Small Cell Lung Cancer. Clin. Cancer Res..

[34] Helin K., Holm K., Niebuhr A., Eiberg H., Tommerup N., Hougaard S., Poulsen H.S., Spang-Thomsen M., Nørgaard P. (1997). Loss of the retinoblastoma protein-related p130 protein in small cell lung carcinoma. Proc. Natl. Acad. Sci. USA.

[35] Miller C.W., Simon K., Aslo A., Kok K., Yokota J., Buys C.H., Terada M., Koeffler H.P. (1992). p53 mutations in human lung tumors. Cancer Res..

[36] Park S., Lee H., Lee B., Lee S.-H., Sun J.-M., Park W.-Y., Ahn J.S., Ahn M.-J., Park K. (2019). DNA Damage Response and Repair Pathway Alteration and Its Association With Tumor Mutation Burden and Platinum-Based Chemotherapy in SCLC. J. Thorac. Oncol..

[37] Byers L.A., Wang J., Nilsson M.B., Fujimoto J., Saintigny P., Yordy J., Giri U., Peyton M., Fan Y.H., Diao L. (2012). Proteomic Profiling Identifies Dysregulated Pathways in Small Cell Lung Cancer and Novel Therapeutic Targets Including PARP1. Cancer Discov..

[38] Pignon J.-P., Tribodet H., Scagliotti G.V., Douillard J.-Y., Shepherd F.A., Stephens R.J., Dunant A., Torri V., Rosell R., Seymour L. (2008). Lung Adjuvant Cisplatin Evaluation: A Pooled Analysis by the LACE Collaborative Group. J. Clin. Oncol..

[39] Cosaert J., Quoix E. (2002). Platinum drugs in the treatment of non-small-cell lung cancer. Br. J. Cancer.

[40] Olaussen K.A., Dunant A., Fouret P., Brambilla E., André F., Haddad V., Taranchon E., Filipits M., Pirker R., Popper H.H. (2006). DNA Repair by ERCC1 in Non–Small-Cell Lung Cancer and Cisplatin-Based Adjuvant Chemotherapy. N. Engl. J. Med..

[41] Vilmar A., Santoni-Rugiu E., Sørensen J. (2010). ERCC1 and histopathology in advanced NSCLC patients randomized in a large multicenter phase III trial. Ann. Oncol..

[42] Simon G.R., Schell M.J., Begum M., Kim J., Chiappori A., Haura E., Antonia S., Bepler G. (2012). Preliminary indication of survival benefit fromERCC1andRRM1-tailored chemotherapy in patients with advanced nonsmall cell lung cancer: Evidence from an individual patient analysis. Cancer.

[43] Okimoto T., Tsubata Y., Tanino R., Nakao M., Hotta T., Hamaguchi M., Hamaguchi S., Araki A., Isobe T. (2021). ERCC1 Is a Predictive Biomarker for Non-small Cell Lung Cancer But Is Antibody-dependent. Anticancer Res..

[44] Malottki K., Popat S., Deeks J.J., Riley R.D., Nicholson A.G., Billingham L. (2016). Problems of variable biomarker evaluation in stratified medicine research—A case study of ERCC1 in non-small-cell lung cancer. Lung Cancer.

[45] Jonsson P., Bandlamudi C., Cheng M.L., Srinivasan P., Chavan S.S., Friedman N.D., Rosen E.Y., Richards A.L., Bouvier N., Selcuklu S.D. (2019). Tumour lineage shapes BRCA-mediated phenotypes. Nature.

[46] Diossy M., Sztupinszki Z., Borcsok J., Krzystanek M., Tisza V., Spisak S., Rusz O., Timar J., Csabai I., Fillinger J. (2021). A subset of lung cancer cases shows robust signs of homologous recombination deficiency associated genomic mutational signatures. NPJ Precis. Oncol..

[47] Huang Z., Xiong G. (2022). BRCA1 expression associated with the prognostic value of platinum-based chemotherapy for stage II–IV non-small cell lung cancer: A meta-analysis. Int. J. Biol. Markers.

[48] Papadaki C., Sfakianaki M., Ioannidis G., Lagoudaki E., Trypaki M., Tryfonidis K., Mavroudis D., Stathopoulos E., Georgoulias V., Souglakos J. (2012). ERCC1 and BRAC1 mRNA Expression Levels in the Primary Tumor Could Predict the Effectiveness of the Second-Line Cisplatin-Based Chemotherapy in Pretreated Patients with Metastatic Non-small Cell Lung Cancer. J. Thorac. Oncol..

[49] Wang L., Meng L., Wang X.-W., Ma G.-Y., Chen J.-H. (2014). Expression of RRM1 and RRM2 as a novel prognostic marker in advanced non-small cell lung cancer receiving chemotherapy. Tumor Biol..

[50] Caiola E., Salles D., Frapolli R., Lupi M., Rotella G., Ronchi A., Garassino M.C., Mattschas N., Colavecchio S., Broggini M. (2015). Base excision repair-mediated resistance to cisplatin in KRAS(G12C) mutant NSCLC cells. Oncotarget.

[51] Zhao W., Hu L., Xu J., Shen H., Hu Z., Ma H., Shu Y., Shao Y., Yin Y. (2013). Polymorphisms in the base excision repair pathway modulate prognosis of platinum-based chemotherapy in advanced non-small cell lung cancer. Cancer Chemother. Pharmacol..

[52] Dai J., Jiang M., He K., Wang H., Chen P., Guo H., Zhao W., Lu H., He Y., Zhou C. (2021). DNA Damage Response and Repair Gene Alterations Increase Tumor Mutational Burden and Promote Poor Prognosis of Advanced Lung Cancer. Front. Oncol..

[53] Owen D.H., Williams T.M., Bertino E.M., Mo X., Webb A., Schweitzer C., Liu T., Roychowdhury S., Timmers C.D., Otterson G.A. (2019). Homologous recombination and DNA repair mutations in patients treated with carboplatin and nab-paclitaxel for metastatic non-small cell lung cancer. Lung Cancer.

[54] Wang Y., Gudikote J., Giri U., Yan J., Deng W., Ye R., Jiang W., Li N., Hobbs B.P., Wang J. (2018). RAD50 Expression Is Associated with Poor Clinical Outcomes after Radiotherapy for Resected Non–small Cell Lung Cancer. Clin. Cancer Res..

[55] Jiang W., Jin G., Cai F., Chen X., Cao N., Zhang X., Liu J., Chen F., Wang F., Dong W. (2019). Extracellular signal-regulated kinase 5 increases radioresistance of lung cancer cells by enhancing the DNA damage response. Exp. Mol. Med..

[56] Zou N., Xie G., Cui T., Srivastava A.K., Qu M., Yang L., Wei S., Zheng Y., Wang Q.-E. (2016). DDB2 increases radioresistance of NSCLC cells by enhancing DNA damage responses. Tumor Biol..

[57] Postel-Vinay S., Vanhecke E., Olaussen K.A., Lord C., Ashworth A., Soria J.-C. (2012). The potential of exploiting DNA-repair defects for optimizing lung cancer treatment. Nat. Rev. Clin. Oncol..

[58] Chae Y.K., Davis A.A., Raparia K., Agte S., Pan A., Mohindra N., Villaflor V., Giles F. (2019). Association of Tumor Mutational Burden With DNA Repair Mutations and Response to Anti–PD-1/PD-L1 Therapy in Non–Small-Cell Lung Cancer. Clin. Lung Cancer.

[59] Chae Y.K., Anker J.F., Oh M.S., Bais P., Namburi S., Agte S., Giles F.J., Chuang J.H. (2019). Mutations in DNA repair genes are associated with increased neoantigen burden and a distinct immunophenotype in lung squamous cell carcinoma. Sci. Rep..

[60] Xiong A., Nie W., Zhou Y., Li C., Gu K., Zhang D., Chen S., Wen F., Zhong H., Han B. (2021). Comutations in DDR Pathways Predict Atezolizumab Response in Non-Small Cell Lung Cancer Patients. Front. Immunol..

[61] Bryant H.E., Schultz N., Thomas H.D., Parker K.M., Flower D., Lopez E., Kyle S., Meuth M., Curtin N.J., Helleday T. (2005). Specific killing of BRCA2-deficient tumours with inhibitors of poly(ADP-ribose) polymerase. Nature.

[62] Rottenberg S., Jaspers J.E., Kersbergen A., Van Der Burg E., Nygren A.O.H., Zander S.A.L., Derksen P.W.B., De Bruin M., Zevenhoven J., Lau A. (2008). High sensitivity of BRCA1-deficient mammary tumors to the PARP inhibitor AZD2281 alone and in combination with platinum drugs. Proc. Natl. Acad. Sci. USA.

[63] Murai J., Feng Y., Yu G.K., Ru Y., Tang S.-W., Shen Y., Pommier Y. (2016). Resistance to PARP inhibitors by SLFN11 inactivation can be overcome by ATR inhibition. Oncotarget.

[64] Ray-Coquard I., Pautier P., Pignata S., Pérol D., González-Martín A., Berger R., Fujiwara K., Vergote I., Colombo N., Mäenpää J. (2019). Olaparib plus Bevacizumab as First-Line Maintenance in Ovarian Cancer. N. Engl. J. Med..

[65] De Bono J., Mateo J., Fizazi K., Saad F., Shore N., Sandhu S., Chi K.N., Sartor O., Agarwal N., Olmos D. (2020). Olaparib for Metastatic Castration-Resistant Prostate Cancer. N. Engl. J. Med..

[66] Swisher E.M., Kwan T.T., Oza A.M., Tinker A.V., Ray-Coquard I., Oaknin A., Coleman R.L., Aghajanian C., Konecny G.E., O’Malley D.M. (2021). Molecular and clinical determinants of response and resistance to rucaparib for recurrent ovarian cancer treatment in ARIEL2 (Parts 1 and 2). Nat. Commun..

[67] Morgan R.D., Clamp A.R., Evans D.G.R., Edmondson R.J., Jayson G.C. (2018). PARP inhibitors in platinum-sensitive high-grade serous ovarian cancer. Cancer Chemother. Pharmacol..

[68] Fennell D.A., Lester J.F., Danson S., Blackhall F.H., Nicolson M., Nixon L.S., Porter C., Gardner G.M., White A., Griffiths G.O. (2020). A randomized phase II trial of olaparib maintenance versus placebo monotherapy in patients with chemosensitive advanced non-small cell lung cancer. J. Clin. Oncol..

[69] Riess J.W., Redman M.W., Wheatley-Price P., Faller B.A., Villaruz L.C., Corum L.R., Gowda A.C., Srkalovic G., Osarogiagbon R.U., Baumgart M.A. (2021). A phase II study of rucaparib in patients with high genomic LOH and/or BRCA 1/2 mutated stage IV non-small cell lung cancer (Lung-MAP Sub-Study, S1900A). J. Clin. Oncol..

[70] Waddington T., Mambetsariev I., Pharaon R., Fricke J., Baroz A.R., Romo H., Ghanem B., Gray S., Salgia R. (2021). Therapeutic Potential of Olaparib in Combination with Pembrolizumab in a Young Patient with a Maternally Inherited BRCA2 Germline Variant: A Research Report. Clin. Lung Cancer.

[71] Jiao S., Xia W., Yamaguchi H., Wei Y., Chen M.K., Hsu J.M., Hsu J.L., Yu W.H., Du Y., Lee H.H. (2017). PARP Inhibitor Upregulates PD-L1 Expression and Enhances Cancer-Associated Immunosuppression. Clin. Cancer Res..

[72] Friedlaender A., Banna G., Malapelle U., Pisapia P., Addeo A. (2019). Next Generation Sequencing and Genetic Alterations in Squamous Cell Lung Carcinoma: Where Are We Today?. Front. Oncol..

[73] Owonikoko T.K., Redman M.W., Byers L.A., Hirsch F.R., Mack P.C., Schwartz L.H., Bradley J.D., Stinchcombe T.E., Leighl N.B., Al Baghdadi T. (2021). Phase 2 Study of Talazoparib in Patients with Homologous Recombination Repair–Deficient Squamous Cell Lung Cancer: Lung-MAP Substudy S1400G. Clin. Lung Cancer.

[74] Paz-Elizur T., Krupsky M., Blumenstein S., Elinger D., Schechtman E., Livneh Z. (2003). DNA Repair Activity for Oxidative Damage and Risk of Lung Cancer. JNCI J. Natl. Cancer Inst..

[75] Jiang Y., Dai H., Li Y., Yin J., Guo S., Lin S.-Y., McGrail D.J. (2019). PARP inhibitors synergize with gemcitabine by potentiating DNA damage in non-small-cell lung cancer. Int. J. Cancer.

[76] Ramalingam S.S., Blais N., Mazieres J., Reck M., Jones C.M., Juhasz E., Urban L., Orlov S., Barlesi F., Kio E. (2017). Randomized, Placebo-Controlled, Phase II Study of Veliparib in Combination with Carboplatin and Paclitaxel for Advanced/Metastatic Non–Small Cell Lung Cancer. Clin. Cancer Res..

[77] Ramalingam S.S., Novello S., Guclu S.Z., Bentsion D., Zvirbule Z., Szilasi M., Bernabe R., Syrigos K., Byers L.A., Clingan P. (2021). Veliparib in Combination With Platinum-Based Chemotherapy for First-Line Treatment of Advanced Squamous Cell Lung Cancer: A Randomized, Multicenter Phase III Study. J. Clin. Oncol..

[78] Wilkerson M.D., Schallheim J.M., Hayes D.N., Roberts P.J., Bastien R.R., Mullins M., Yin X., Miller C.R., Thorne L.B., Geiersbach K.B. (2013). Prediction of Lung Cancer Histological Types by RT-qPCR Gene Expression in FFPE Specimens. J. Mol. Diagn..

[79] Govindan R., Lind M., Insa A., Khan S.A., Uskov D., Tafreshi A., Guclu S., Bar J., Kato T., Lee K.H. (2022). Veliparib Plus Carboplatin and Paclitaxel Versus Investigator’s Choice of Standard Chemotherapy in Patients With Advanced Non–Squamous Non–Small Cell Lung Cancer. Clin. Lung Cancer.

[80] Li A., Yi M., Qin S., Chu Q., Luo S., Wu K. (2019). Prospects for combining immune checkpoint blockade with PARP inhibition. J. Hematol. Oncol..

[81] Clarke J.M., Patel J.D., Robert F., Kio E.A., Thara E., Camidge D.R., Dunbar M., Nuthalapati S., Dinh M.H., Bach B.A. (2021). Veliparib and nivolumab in combination with platinum doublet chemotherapy in patients with metastatic or advanced non-small cell lung cancer: A phase 1 dose escalation study. Lung Cancer.

[82] Fumet J.-D., Limagne E., Thibaudin M., Truntzer C., Bertaut A., Rederstorff E., Ghiringhelli F. (2020). Precision medicine phase II study evaluating the efficacy of a double immunotherapy by durvalumab and tremelimumab combined with olaparib in patients with solid cancers and carriers of homologous recombination repair genes mutation in response or stable after olaparib treatment. BMC Cancer.

[83] Passiglia F., Bironzo P., Righi L., Listì A., Arizio F., Novello S., Volante M., Scagliotti G.V. (2021). A Prospective Phase II Single-arm Study of Niraparib Plus Dostarlimab in Patients With Advanced Non–small-cell Lung Cancer and/or Malignant Pleural Mesothelioma, Positive for PD-L1 Expression and Germline or Somatic Mutations in the DNA Repair Genes: Rationale and Study Design. Clin. Lung Cancer.

[84] Garcia-Campelo R., Arrieta O., Massuti B., Rodriguez-Abreu D., Granados A.L.O., Majem M., Vicente D., Lianes P., Bosch-Barrera J., Insa A. (2020). Combination of gefitinib and olaparib versus gefitinib alone in EGFR mutant non-small-cell lung cancer (NSCLC): A multicenter, randomized phase II study (GOAL). Lung Cancer.

[85] de Haan R., van der Heuvel M.M., van Diessen J., Peulen H.M., van Werkhoven E., de Langen A.J., Lalezari F., Pluim D., Verwijs-Janssen M., Vens C. (2021). Phase I and Pharmacologic Study of Olaparib in Combination with High-dose Radiotherapy with and without Concurrent Cisplatin for Non–Small Cell Lung Cancer. Clin. Cancer Res..

[86] Ramalingam S.S., Thara E., Awad M.M., Dowlati A., Haque B., Stinchcombe T.E., Dy G.K., Spigel D.R., Lu S., Singh N.I. (2022). JASPER: Phase 2 trial of first-line niraparib plus pembrolizumab in patients with advanced non–small cell lung cancer. Cancer.

[87] Chabot P., Hsia T.-C., Ryu J.-S., Gorbunova V., Belda-Iniesta C., Ball D., Kio E., Mehta M., Papp K., Qin Q. (2017). Veliparib in combination with whole-brain radiation therapy for patients with brain metastases from non-small cell lung cancer: Results of a randomized, global, placebo-controlled study. J. Neuro-Oncol..

[88] Marzio A., Kurz E., Sahni J.M., Di Feo G., Puccini J., Jiang S., Hirsch C.A., Arbini A.A., Wu W.L., Pass H.I. (2022). EMSY inhibits homologous recombination repair and the interferon response, promoting lung cancer immune evasion. Cell.

[89] Vendetti F.P., Lau A., Schamus S., Conrads T.P., O’Connor M.J., Bakkenist C.J. (2015). The orally active and bioavailable ATR kinase inhibitor AZD6738 potentiates the anti-tumor effects of cisplatin to resolve ATM-deficient non-small cell lung cancer in vivo. Oncotarget.

[90] Dunne V., Ghita M., Small D.M., Coffey C.B., Weldon S., Taggart C.C., Osman S.O., McGarry C.K., Prise K.M., Hanna G.G. (2017). Inhibition of ataxia telangiectasia related-3 (ATR) improves therapeutic index in preclinical models of non-small cell lung cancer (NSCLC) radiotherapy. Radiother. Oncol..

[91] Yap T.A., Krebs M.G., Postel-Vinay S., Bang Y.J., El-Khoueiry A., Abida W., Harrington K., Sundar R., Carter L., Castanon-Alvarez E. (2016). Phase I modular study of AZD6738, a novel oral, potent and selective ataxia telangiectasia Rad3-related (ATR) inhibitor in combination (combo) with carboplatin, olaparib or durvalumab in patients (pts) with advanced cancers. Eur. J. Cancer.

[92] Sherr C.J., McCormick F. (2002). The RB and p53 pathways in cancer. Cancer Cell.

[93] Lok B.H., Gardner E.E., Schneeberger V.E., Ni A., Desmeules P., Rekhtman N., De Stanchina E., Teicher B.A., Riaz N., Powell S.N. (2017). PARP Inhibitor Activity Correlates with SLFN11 Expression and Demonstrates Synergy with Temozolomide in Small Cell Lung Cancer. Clin. Cancer Res..

[94] Pietanza M.C., Waqar S.N., Krug L.M., Dowlati A., Hann C.L., Chiappori A., Owonikoko T.K., Woo K.M., Cardnell R.J., Fujimoto J. (2018). Randomized, Double-Blind, Phase II Study of Temozolomide in Combination With Either Veliparib or Placebo in Patients With Relapsed-Sensitive or Refractory Small-Cell Lung Cancer. J. Clin. Oncol..

[95] Gay C.M., Stewart C.A., Park E.M., Diao L., Groves S.M., Heeke S., Nabet B.Y., Fujimoto J., Solis L.M., Lu W. (2021). Patterns of transcription factor programs and immune pathway activation define four major subtypes of SCLC with distinct therapeutic vulnerabilities. Cancer Cell.

[96] Cardnell R.J., Feng Y., Diao L., Fan Y.-H., Masrorpour F., Wang J., Shen Y., Mills G.B., Minna J.D., Heymach J.V. (2013). Proteomic Markers of DNA Repair and PI3K Pathway Activation Predict Response to the PARP Inhibitor BMN 673 in Small Cell Lung Cancer. Clin. Cancer Res..

[97] Sen T., Tong P., Stewart C.A., Cristea S., Valliani A., Shames D.S., Redwood A.B., Fan Y.H., Li L., Glisson B.S. (2017). CHK1 Inhibition in Small-Cell Lung Cancer Produces Single-Agent Activity in Biomarker-Defined Disease Subsets and Combination Activity with Cisplatin or Olaparib. Cancer Res..

[98] de Bono J., Ramanathan R.K., Mina L., Chugh R., Glaspy J., Rafii S., Kaye S., Sachdev J., Heymach J., Smith D.C. (2017). Phase I, Dose-Escalation, Two-Part Trial of the PARP Inhibitor Talazoparib in Patients with Advanced Germline *BRCA1/2* Mutations and Selected Sporadic Cancers. Cancer Discov..

[99] Woll P., Gaunt P., Danson S., Steele N., Ahmed S., Mulatero C., Shah R., Bhosle J., Hodgkinson E., Watkins B. (2022). Olaparib as maintenance treatment in patients with chemosensitive small cell lung cancer (STOMP): A randomised, double-blind, placebo-controlled phase II trial. Lung Cancer.

[100] Atrafi F., Groen H.J., Byers L.A., Garralda E., Lolkema M.P., Sangha R.S., Viteri S., Chae Y.K., Camidge D.R., Gabrail N.Y. (2019). A Phase I Dose-Escalation Study of Veliparib Combined with Carboplatin and Etoposide in Patients with Extensive-Stage Small Cell Lung Cancer and Other Solid Tumors. Clin. Cancer Res..

[101] Owonikoko T.K., Dahlberg S.E., Sica G.L., Wagner L.I., Wade J.L., Srkalovic G., Lash B.W., Leach J.W., Leal T.B., Aggarwal C. (2019). Randomized Phase II Trial of Cisplatin and Etoposide in Combination With Veliparib or Placebo for Extensive-Stage Small-Cell Lung Cancer: ECOG-ACRIN 2511 Study. J. Clin. Oncol..

[102] Farago A.F., Yeap B.Y., Stanzione M., Hung Y.P., Heist R.S., Marcoux J.P., Zhong J., Rangachari D., Barbie D.A., Phat S. (2019). Combination Olaparib and Temozolomide in Relapsed Small-Cell Lung Cancer. Cancer Discov..

[103] Laird J.H., Lok B.H., Ma J., Bell A., de Stanchina E., Poirier J.T., Rudin C.M. (2018). Talazoparib Is a Potent Radiosensitizer in Small Cell Lung Cancer Cell Lines and Xenografts. Clin. Cancer Res..

[104] Foy V., Schenk M.W., Baker K., Gomes F., Lallo A., Frese K.K., Forster M., Dive C., Blackhall F. (2017). Targeting DNA damage in SCLC. Lung Cancer.

[105] Lallo A., Frese K.K., Morrow C.J., Sloane R., Gulati S., Schenk M.W., Trapani F., Simms N., Galvin M., Brown S. (2018). The Combination of the PARP Inhibitor Olaparib and the WEE1 Inhibitor AZD1775 as a New Therapeutic Option for Small Cell Lung Cancer. Clin. Cancer Res..

[106] Gardner E.E., Lok B.H., Schneeberger V.E., Desmeules P., Miles L.A., Arnold P.K., Ni A., Khodos I., de Stanchina E., Nguyen T. (2017). Chemosensitive Relapse in Small Cell Lung Cancer Proceeds through an EZH2-SLFN11 Axis. Cancer Cell.

[107] Barayan R., Ran X., Lok B.H. (2020). PARP inhibitors for small cell lung cancer and their potential for integration into current treatment approaches. J. Thorac. Dis..

[108] Liu J., Barry W., Birrer M., Lee J.-M., Buckanovich R., Fleming G., Rimel B., Buss M., Nattam S., Hurteau J. (2019). Overall survival and updated progression-free survival outcomes in a randomized phase II study of combination cediranib and olaparib versus olaparib in relapsed platinum-sensitive ovarian cancer. Ann. Oncol..

[109] Kaplan A.R., Gueble S.E., Liu Y., Oeck S., Kim H., Yun Z., Glazer P.M. (2019). Cediranib suppresses homology-directed DNA repair through down-regulation of BRCA1/2 and RAD. Sci. Transl. Med..

[110] Cardnell R.J., Feng Y., Mukherjee S., Diao L., Tong P., Stewart C.A., Masrorpour F., Fan Y., Nilsson M., Shen Y. (2016). Activation of the PI3K/mTOR Pathway following PARP Inhibition in Small Cell Lung Cancer. PLoS ONE.

[111] Shen J., Zhao W., Ju Z., Wang L., Peng Y., Labrie M., Yap T.A., Mills G.B., Peng G. (2019). PARPi Triggers the STING-Dependent Immune Response and Enhances the Therapeutic Efficacy of Immune Checkpoint Blockade Independent of BRCAness. Cancer Res..

[112] Thomas A., Vilimas R., Trindade C., Erwin-Cohen R., Roper N., Xi L., Krishnasamy V., Levy E., Mammen A., Nichols S. (2019). Durvalumab in Combination with Olaparib in Patients with Relapsed SCLC: Results from a Phase II Study. J. Thorac. Oncol..

[113] Peyraud F., Italiano A. (2020). Combined PARP Inhibition and Immune Checkpoint Therapy in Solid Tumors. Cancers.

[114] Wu Z., Tao H., Zhang S., Wang X., Ma J., Li R., Liu Z., Wang J., Cui P., Chen S. (2021). Efficacy and safety of anti-PD-1-based therapy in combination with PARP inhibitors for patients with advanced solid tumors in a real-world setting. Cancer Immunol. Immunother..

