# Supplementary material for: The Prognostic and Therapeutic Potential of DNA Damage Repair Pathway Alterations and Homologous Recombination Deficiency in Lung Cancer
Source: Cancers (Basel). 2022 Oct 28;14(21):5305. doi: 10.3390/cancers14215305 (PMC9654807; doi:10.3390/cancers14215305)
Supplement: Supplementary file 1 [file cancers-14-05305-s001.zip › cancers-1965345-supplementary.pdf]

Table S1. Select clinical trials of DNA-damage repair targeted agents in clinical trials in solid tumors including non-small cell lung cancer.

Clinical Trial	Medication	DNA-Damage Repair Target by Novel Medication	Tumor Type	Inclusion Criteria	Exclusion Criteria	Selective Biomarkers	End Point
NCT04216316	Addition of Berzosertib (M6620, VX-970) to carboplatin and gemcitabine and pembrolizumab Phase IB, II	ATR inhibitor	Squamous NSCLC	Stage IV disease, Age ≥ 18, ECOG 0-1	Prior chemo/immuno therapy for advanced disease, Autoimmune disease	ATM mutation	*Primary:* RP2D PFS *Secondary:* PFS (in ATM-deficient), OR, worse grade of adverse events
NCT02589522	Berzosertib (M6620,(VX-970)) with WBRT Phase I	ATR inhibitor	NSCLC, neuroendocrine, small cell lung cancer	Stage IV disease, CNS disease eligible for palliative WBRT, ECOG 0-1 Life expectancy > 2 months	Patients with 1-3 brain mets, eligible for SRS/SRT, greater than 1cm mid-line shift	Germline DDR defects excluded	*Primary:* Incidence of dose limiting toxicity *Secondary:* icPFS, OS, QOL, RRR
NCT05450692	Ceralasertib (AZD6738) plus durvalumab versus docetaxel Phase III	ATR inhibitor	NSCLC	Locally advanced or metastatic disease, PD on last line of treatment, life expectancy > 3 months	Persistent toxicity from prior treatment, history of autoimmunedis order	EGFR/ALK wild type	*Primary:* OS *Secondary:* PFS, ORR, DoR, TTR, DCR, PFS2, TTD of QoL, AEs
NCT02264678	Ascending dosing of ceralasertib in combination with chemo or novel agents Phase I	ATR inhibitor	NSCLC, H&N SCC, gastric, breast, ovarian ca	Presence of solid tumor not considered appropriate for further standard treatment	Prior exposure to ATM inhibitors, prior treatment with PARPi	ATM	*Primary:* AE, sAE *Secondary:* Cmax, PFS, ORR
NCT03334617	Umbrella study of novel agents (ceralasertib) +Durvalumab Phase II	ATR inhibitor	NSCLC	Stage IV, progressed on anti PD1/PD-L1, life expectancy ≥ 3 months	History of primary immunodeficiency or autoimmune disorders, CNS disease	EGFR mutation negative ALK fusions negative ROS1, BRAF, MET, RET negative	*Primary:* ORR *Secondary:* DCR, DoR, PFS, OS
NCT03833440	Precision Immuno-Oncology for Advanced Non-small Cell Lung Cancer Patients	ATR Inhibitor	NSCLC	ECOG 0-1, progression after second- or third-line PD-1, PDL-1 inihibitor	Symptomatic or untreated CNS disease, prior autoimmune disease	N/A	*Primary:* 12w disease control rate *Secondary:* ORR, PFS, OS, duration of response

	With PD-1 ICI Resistance Phase 2						
NCT02588105	Safety and efficacy of AZD0156 at increase alone or in combination with other anti-cancer treatment in patients with advanced cancer Phase I	ATM inhibitor	Solid tumors	Locally advanced or metastatic cancer, refractory to standard therapy, ECOG 0-1,	Prior treatment with ATM inhibitor, poor lung reserve, symptomatic CNS disease	N/A	*Primary:* AE rate *Secondary:* OS, MTD, RAC, CLR,
NCT04550104	Platform of novel agents (AZD0156) in combination with radiotherapy Phase I	ATM inhibitor	NSCLC	Stage IIB, IIIA/IIIB planned to receive curative RT, ECOG 1-2, life expectancy > 6 months	Progression during induction chemotherapy, prior thoracic RT, treatment with pneumotoxic drugs	N/A	*Primary:* Dose limiting toxicities *Secondary:* OS, PFS, QLQ-C30, ORR, treatment compliance
NCT00006464	UCN-01 and Cisplatin Phase I	Protein Kinase C inhibitor	Solid tumors	Advanced or metastatic disease without standard curative or palliative measures, ECOG 0-2	Pulmonary dysfunction, NYHA III-IV CHF, DM	N/A	*Primary:* Dose-limiting toxicities of cisplatin w/ UCN-01 *Secondary:* ORR, peak plasma concentration
NCT00004059	UCN-01 and Fluorouracil Phase I	Protein Kinase C inhibitor	Solid tumors	Advanced or metastatic disease without standard curative or palliative measures, Karnofsky 60-100%	CAD, DLCO <60% of predicted, CNS disease	N/A	*Primary:* Dose-limiting toxicities of 5-FU w/ UCN-01 *Secondary:* ORR, peak plasma concentration
NCT00047242	UCN-01 and Irinotecan Phase I	Protein Kinase C inhibitor	Solid tumors	Advanced or metastatic disease without standard curative or palliative measures ECOG 0-2 Life expectance > 3 months	CAD, LVEF < 50%, DLC O < 75%	N/A	*Primary:* Maximum tolerated dose of UCN-01 + irinotecan *Secondary:* ORR, peak plasma concentration
NCT00551512	CBP501 and Cisplatin Phase I	CHEK1/2 Inhibitor	Solid tumors	Advanced or metastatic disease without standard curative or	Prior chemotherapy with nitrosoureas or high dose carboplatin,	N/A	*Primary:* Dose limiting toxicity *Secondary:* AE, sAE, ORR, PFS

				palliative measures	active CNS disease		
NCT00942825	First line triplet combination (CBP501, pemetrexed, and cisplatin) Phase II	CHEK1/2 Inhibitor	Non-squamous NSCLC	Locally advanced or metastatic disease, ECOG 0-1, Life expectance 0-1	Symptomatic CHF, peripheral neuropathy > grade 1, symptomatic CNS disease	N/A	*Primary:* PFS *Secondary:* ORR, OS, AE, sAE
NCT03113188	CBP501, Cisplatin, Nivolumab Phase IB	CHEK1/2 Inhibitor	Solid Tumor	Locally advanced or metastatic disease, prior treatment, ECOG 0-1, Life expectance > 3 months	Prior chemotherapy with nitrosoureas, peripheral neuropathy > grade 1, active CNS disease	N/A	*Primary:* RP2D *Secondary:* ORR
NCT00413686	AZD7762 alone and in combination with Gemcitabine Phase I	CHEK2 inhibitor	Solid Tumor	Locally advanced or metastatic disease without standard curative or palliative therapy, ECOG 0-1	NYHA II-IV CHD, prior anthracycline, impaired renal or liver function	N/A	*Primary:* Safety and tolerability *Secondary:* Single and combination dose PK
NCT00415636	Safety and tolerability of IC83/LY2603618 Phase 1	CHEK1 inhibitor	Solid tumor	At least one lesion that can be evaluated by RECIST, life expectancy > 3 months	CNS disease, pregnancy	N/A	*Primary:* AEs *Secondary:* Cmax, percentage of patients with best overall response,
NCT00988858	IC83/LY2603618 in combination with Pemetrexed	CHEK1 inhibitor	NSCLC	Advanced or metastatic disease that has progressed on prior treatment	Pregnancy, prior treatment with pemetrexed	N/A	*Primary:* ORR, percentage achieving CR, PR. *Secondary:* PFS, duration of response, LCSS, Cmax
NCT01139775	LY2603618 in Combination with Pemetrexed and Cisplatin Phase I/II	CHEK1 inhibitor	Non-squamous NSCLC	Eligible for first line treatment with platinum doublet, ECOG 0-1	CNS metastasis, serious co-morbidities, third space fluid collection	N/A	*Primary:* Recommended phase 2 dose, PFS *Secondary:* OS, ORR, Cmax, LCSS, mortality
NCT00437203	PF-00477736 in combination with gemcitabine Phase I	CHEK1 inhibitor	Solid tumors	Advanced/metastatic disease refractory to standard therapy, ECOG 0-1	Prior treatment with metastasis, uncontrolled CNS disease,	N/A	*Primary:* MTD with gemcitabine *Secondary:* OR, Cmax,

NCT02860780	Prexasertib (LY2606368) in combination with Ramlimetinib Phase I	CHEK1/2 inhibitor	Solid tumor	Advanced or metastatic disease, able to swallow tablets	Active infection, active CNS disease, autoimmune disorders.	KRAS and or/BRAF mutations	*Primary:* MTD *Secondary:* PK – Cmax, AUC, BOR, DCR, DOR, PFs
NCT02087176	AZD1775 + docetaxel vs placebo + docetaxel Phase II	WEE1 inhibitor	NSCLC	Failure of one platinum-based doublet, ECOG 0-1	Known CNS disease, active infection, NYHA II-IV CHF	N/A	*Primary:* ORR *Secondary:* Pharmacokinetic profile
NCT02087241	Carboplatin and Pemetrexed with or without AZD1775 Phase II	WEE1 inhibitor	NSCLC	Recurrent of Stage IV disease, egfr/alk mut patients prior treatment w/ inhibitor ECOG 0-1	Known CNS disease, NYHA II-IV CHF, presence of other active cancers	EGFR mutation, ALK mutation	*Primary:* PFS *Secondary:* ORR, Disease control rate, duration of response, OS
NCT02513563	AZD1775 plus carboplatin-pacitaxel Phase II	WEE1 inhibitor	SquamousCLC	Advanced/metastatic disease without curative options, ECOG 0-1	Progressive/symptomatic brain mets, use of platinum or paclitaxel for stage IV disease NYHA II-IV CHF	N/A	*Primary:* PFS *Secondary:* OS, OR, SD, DCR
NCT01748825	AZD1775 for Advanced Solid Tumors Phase I	WEE1 inhibitor	Solid tumor	Advanced or metastatic disease without or refractory to standard therapy, ECOG 0-1, Life expectancy > 3 months	Known CNS disease, uncontrolled illness, pregnant women	*N/A*	*Primary:* Safety and tolerability, pharmacokinetics *Secondary:* ORR
NCT02511795	AZD1775 combined with olaparib Phase I	WEE1 inhibitor	Solid tumor	Advanced or metastatic disease without standard treatment, ECOG 0-1	Prior treatment with PARP inhibitor, persistent Grade > 1 toxicity from prior cancer therapy, known CNS disease	N/A	*Primary:* DLTs, TEAEs *Secondary:* Cmax, AUC, ORR, PFS, OS, DCR
